# 3′ UTR lengthening as a novel mechanism in regulating cellular senescence

**DOI:** 10.1101/gr.224451.117

**Published:** 2018-03

**Authors:** Meng Chen, Guoliang Lyu, Miao Han, Hongbo Nie, Ting Shen, Wei Chen, Yichi Niu, Yifan Song, Xueping Li, Huan Li, Xinyu Chen, Ziyue Wang, Zheng Xia, Wei Li, Xiao-Li Tian, Chen Ding, Jun Gu, Yufang Zheng, Xinhua Liu, Jinfeng Hu, Gang Wei, Wei Tao, Ting Ni

**Affiliations:** 1 State Key Laboratory of Genetic Engineering and Ministry of Education (MOE) Key Laboratory of Contemporary Anthropology, Collaborative Innovation Center of Genetics and Development, School of Life Sciences and Huashan Hospital, Fudan University, Shanghai, 200438 China;; 2 MOE Key Laboratory of Cell Proliferation and Differentiation, School of Life Sciences, Peking University, Beijing, 100871 China;; 3 Division of Biostatistics, Dan L. Duncan Cancer Center and Department of Molecular and Cellular Biology, Baylor College of Medicine, Houston, Texas 77030, USA;; 4 Human Aging Research Institute and School of Life Sciences, Nanchang University, Nanchang, 330031 China;; 5 State Key Laboratory of Genetic Engineering, Collaborative Innovation Center of Genetics and Development, School of Life Sciences, Fudan University, Shanghai, 200438 China;; 6 School of Pharmacy, Fudan University, Shanghai, 201203 China

## Abstract

Cellular senescence has been viewed as a tumor suppression mechanism and also as a contributor to individual aging. Widespread shortening of 3′ untranslated regions (3′ UTRs) in messenger RNAs (mRNAs) by alternative polyadenylation (APA) has recently been discovered in cancer cells. However, the role of APA in the process of cellular senescence remains elusive. Here, we found that hundreds of genes in senescent cells tended to use distal poly(A) (pA) sites, leading to a global lengthening of 3′ UTRs and reduced gene expression. Genes that harbor longer 3′ UTRs in senescent cells were enriched in senescence-related pathways. *Rras2*, a member of the Ras superfamily that participates in multiple signal transduction pathways, preferred longer 3′ UTR usage and exhibited decreased expression in senescent cells. Depletion of *Rras2* promoted senescence, while rescue of *Rras2* reversed senescence-associated phenotypes. Mechanistically, splicing factor TRA2B bound to a core “AGAA” motif located in the alternative 3′ UTR of *Rras2*, thereby reducing the RRAS2 protein level and causing senescence. Both proximal and distal poly(A) signals showed strong sequence conservation, highlighting the vital role of APA regulation during evolution. Our results revealed APA as a novel mechanism in regulating cellular senescence.

Cellular senescence was originally described as a process that limits the proliferation of cultured human cells. After extensive proliferation, senescence occurs because of telomere shortening and loss in the absence of endogenous telomerase activity (Hayflick and Moorhead 1961; Olovnikov 1996). In addition to telomere erosion, many stimuli and stresses can cause cellular senescence, including DNA double-strand breaks, strong mitogenic signals, oxidative stress, and ectopic expression of cyclin-dependent kinase inhibitors (CDKIs) (Xue et al. 2004; Rodier and Campisi 2011; Li et al. 2013). Numerous morphological and molecular markers of senescent cells have been identified in recent decades, which include a flattened and enlarged cell morphology, increased senescence-associated β-galactosidase (SA-β-gal) activity, reduced proliferation rate, and expression of tumor suppressors, cell cycle inhibitors, and DNA damage markers (Dimri et al. 1995; Busuttil et al. 2003; Lopez-Otin et al. 2013; Munoz-Espin and Serrano 2014). Cellular senescence is viewed as an important mechanism for preventing cancer (Campisi et al. 2001). It is also involved in normal embryonic development and tissue damage (Munoz-Espin et al. 2013; Storer et al. 2013; Munoz-Espin and Serrano 2014). Removing senescent cells expands the healthy lifespan of mice (Baker et al. 2016). These results demonstrate the significance of cellular senescence.

A number of studies have shown that dramatic changes in the transcriptome and/or proteome accompany the phenotypic alterations of senescent cells (Kim et al. 2013b; Mazin et al. 2013; Waldera-Lupa et al. 2014; Wei et al. 2015) and that the development of senescence-associated phenotypes can be regulated by stage-specific gene expression modules (Kim et al. 2013b). Therefore, understanding the regulation of gene expression and the corresponding regulatory networks is crucial to dissecting the mechanism of cellular senescence. Alternative polyadenylation (APA) is recognized as a crucial contributor to the regulation of mammalian gene expression (Di Giammartino et al. 2011; Elkon et al. 2013; Chen et al. 2017; Tian and Manley 2017). Cleavage and polyadenylation of nascent RNA is essential for maturation of the vast majority of eukaryotic mRNAs and determines the length of 3′ UTRs (Sachs 1990). The process requires several *cis*-acting RNA elements and dozens of *trans*-factors (Millevoi and Vagner 2010). The key *cis*-element is a six-nucleotide (nt) motif (termed the poly[A] signal or PAS), the canonical form of which is “AAUAAA.” The PAS determines recognition and cleavage by the 3′ end-processing machinery (Proudfoot and Brownlee 1976). The mammalian 3′ end-processing machinery contains several subcomplexes as well as additional accessory factors which mediate the precise processing of mRNA precursors (pre-mRNAs) together (Di Giammartino et al. 2011; Elkon et al. 2013).

With the increasing application of high-throughput sequencing technologies, genome-wide studies indicate that most eukaryotic mRNA genes have multiple polyadenylation (pA) sites (Tian et al. 2005; Wang et al. 2008; Zheng and Tian 2014). Alternative pA sites can reside in the 3′-most exon or upstream regions and can give rise to multiple mRNA transcripts that contain different coding sequences, 3′ UTRs, or both (Di Giammartino et al. 2011; Elkon et al. 2013). Importantly, both microRNAs (miRNAs) and RNA binding proteins (RBPs) targeting 3′ UTRs are able to regulate translational efficiency, degradation, and subcellular localization of mRNA or protein (Di Giammartino et al. 2011; Berkovits and Mayr 2015; Tian and Manley 2017). It is well known that APA plays important roles in a wide range of biological processes such as cell differentiation (Ji et al. 2009; Mangone et al. 2010; Hilgers et al. 2011; Li et al. 2012; Ulitsky et al. 2012; Fu et al. 2016; Hu et al. 2017), cell proliferation (Sandberg et al. 2008; Elkon et al. 2012; Hoffman et al. 2016), cell/tissue identity (Zhang et al. 2005; Derti et al. 2012; Smibert et al. 2012; Ni et al. 2013), and carcinogenesis (Mayr and Bartel 2009; Fu et al. 2011; Lin et al. 2012; Xia et al. 2014). However, whether APA is involved in senescence-associated gene expression and contributes to cellular senescence remains to be answered.

We therefore examined the potential role and possible mechanism of APA in cellular senescence by applying our polyadenylation sequencing (PA-seq) approach (Ni et al. 2013) in two cellular senescence models, the passage of mouse embryonic fibroblasts (MEFs) and aortic vascular smooth muscle cells of rats (rVSMCs) at different ages.

## Results

### Global lengthening of 3′ UTRs couples with decreased gene expression in senescent cells

**Figure 1.**
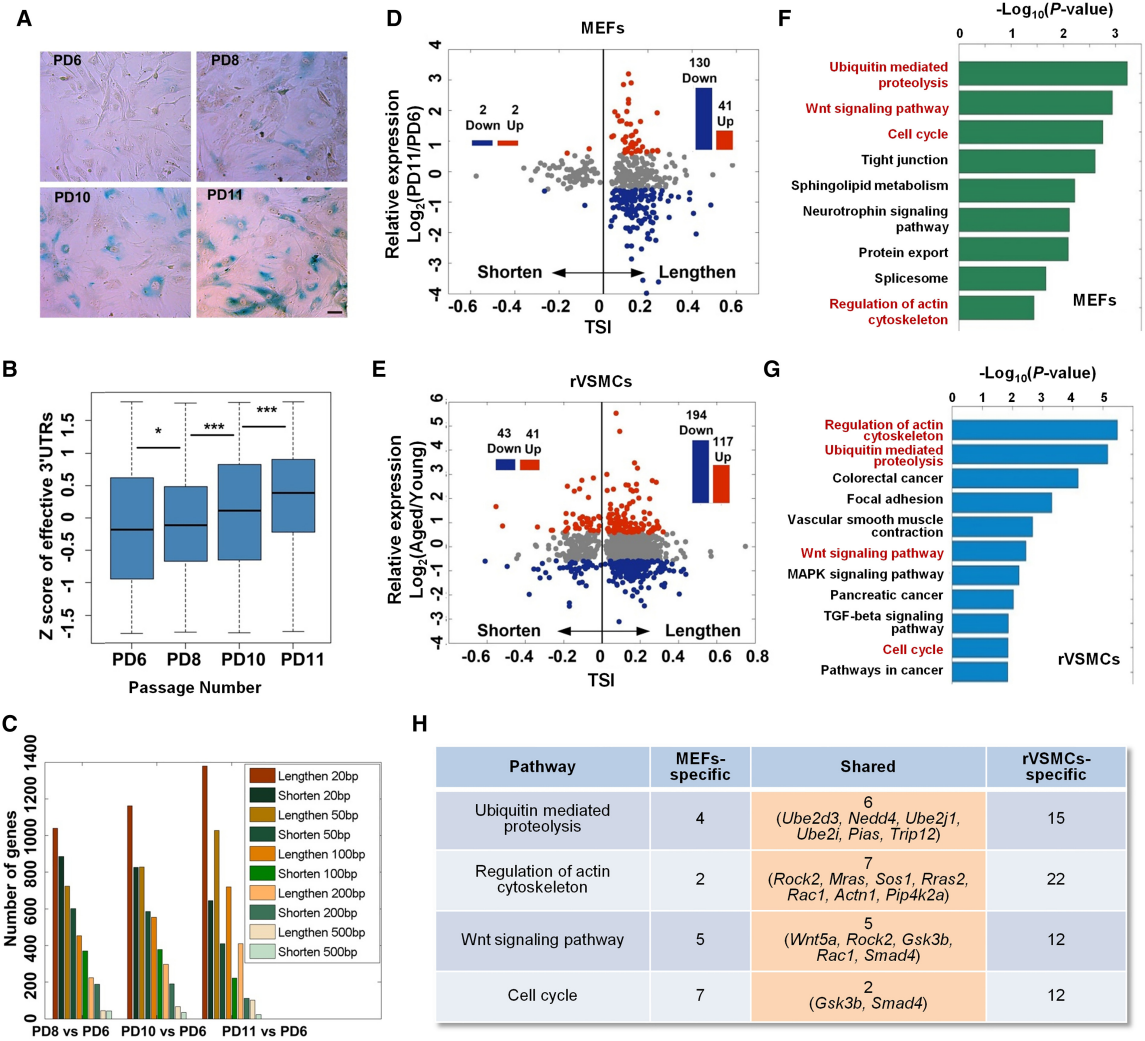
Global lengthening of 3′ UTRs in senescent cells. (*A*) SA-β-gal staining for MEFs of PD6, PD8, PD10, and PD11 passages. (*B*) Box plot for *Z*-score transformed effective 3′ UTRs across PD6, PD8, PD10, and PD11 MEFs. (*C*) Number of genes with lengthened and shortened effective 3′ UTRs. (*D*) Scatter plot between TSI and expression changes for genes with significantly longer or shorter 3′ UTRs in senescent MEFs (PD11 vs. PD6). TSI denotes tandem UTR isoform switch index (see Methods). The red and blue histograms represent up- and down-regulation (1.5-fold) in senescent cells, respectively. (*E*) Scatter plot between TSI and expression changes for genes with significantly longer or shorter 3′ UTRs in rVSMCs (old vs. young), similar to that in *D*. (*F*,*G*) Pathways significantly enriched (*P* < 0.01, Fisher's exact test) for genes tended to use distal pA sites (TSI > 0 in *D* and *E*) in senescent MEFs (*F*) and VSMCs derived from old rats (*G*). The shared senescence-related pathways are marked in red text. (*H*) Overlap of genes in the shared pathways between senescent MEFs and rat VSMCs. The number of genes is shown in each grid and shared gene names are in brackets.

To determine whether APA plays a role during cellular senescence, we first used MEFs undergoing replicative senescence in vitro and having population doubling (PD) times of 6, 8, 10, and 11 passages (Fig. 1A; Supplemental Fig. S1; Dimri et al. 1995; Parrinello et al. 2003; Tian and Li 2014; Tigges et al. 2014). We applied RNA sequencing (RNA-seq) and PA-seq (Ni et al. 2013) to discern the relationship between gene expression and 3′ UTR length patterns in these cells. After confirming the reliability of called pA sites, such as genomic location distribution, poly(A) signal enrichment, and overlap with polyA_DB (Supplemental Figs. S2, S3; Supplemental Table S1), the effective 3′ UTR length (or weighted mean of 3′ UTR length) was used to estimate the relative trend of pA site usage and changes in 3′ UTR length according to our previous method, which took pA site location, the distance to a stop codon, and tag number into consideration (Ni et al. 2013). Effective 3′ UTRs showed a global lengthening trend during MEFs senescence (Fig. 1B), indicating a tendency to use the distal pA sites. To further evaluate the changes in 3′ UTR length at the level of individual genes, we compared effective 3′ UTR length in later passaged (PD8, PD10, and PD11) cells with that in the earlier passaged (PD6) cells using different cut-offs. Cells of later passages always had many more genes with a longer 3′ UTR compared with cells of the earlier passage (Fig. 1C). Moreover, the number of genes with a lengthened 3′ UTR gradually increased from PD8 to PD11, while the number of genes with a shortened 3′ UTR continuously decreased (Fig. 1C). The same trend of global 3′ UTR lengthening was further confirmed by adopting a different methodology, the relative usage of distal polyadenylation sites (RUD) index (Ji et al. 2011), using separate RNA-seq data from the same MEFs used in PA-seq (Supplemental Figs. S4, S5; Supplemental Table S2). Biological replicates of PD11 MEFs had more genes with a longer 3′ UTR compared with those of PD6 MEFs (Supplemental Fig. S6; Supplemental Table S3). Together, these results demonstrate the global lengthening of 3′ UTRs in senescent MEFs.

To determine whether cell cycle affects the length of 3′ UTRs during senescence, PD6 MEF cells were subjected to serum starvation to drive them into G0 phase (Gustincich and Schneider 1993). The global pattern of 3′ UTR length in G0 cells was most similar to that in PD6 compared with other passages (Supplemental Figs. S5A, S7; Supplemental Tables S1, S2), implying that the global lengthening of 3′ UTRs resulted from senescence rather than from cell cycle alterations.

A longer 3′ UTR region could provide more opportunities for regulation by miRNAs and/or RBPs, which would influence mRNA and/or protein abundance at the post-transcriptional level (Mayr and Bartel 2009; Zheng and Tian 2014). In line with this hypothesis, a global decline in gene expression was observed for genes preferring distal pA sites during senescence (Supplemental Fig. S8A,C–E). In contrast, control genes with a single pA site or favoring proximal pA sites during senescence did not show such a trend (Supplemental Fig. S8B,F,G). Meanwhile, changes in APA and gene expression at the individual gene level were also analyzed by comparing PA-seq data from PD11 and PD6 MEFs using TSI (tandem 3′ UTR isoform switch index), a higher value of which indicates 3′ UTR lengthening (Fu et al. 2011; Li et al. 2012). Genes with a significantly lengthened 3′ UTR outnumbered those containing a shortened one (Fig. 1C,D; Supplemental Fig. S9). Within 3′ UTR lengthened genes, more genes showed reduced expression than elevated expression (*P* < 5.6 × 10^−12^, binomial test) (Fig. 1D, right half), while no such difference was observed for genes with shorter 3′ UTRs (Fig. 1D, left half). These data suggest that global 3′ UTR lengthening is associated with decreased gene expression during cellular senescence.

To explore whether APA-induced global lengthening of 3′ UTRs occurred in other senescence systems, we performed PA-seq in VSMCs derived from young and aged rats (Supplemental Table S4). The results showed that senescent rVSMCs and MEFs had a similar ratio between numbers of genes with longer and shorter 3′ UTRs (Supplemental Fig. S9). A significant overlap of genes with longer 3′ UTR usage was observed in these two senescence models (Supplemental Figs. S10, S11). The observation that more genes with lengthened 3′ UTRs tended to be down-regulated was also verified in rVSMCs (*P* < 1.5 × 10^−5^, binomial test) (Fig. 1E). Together, the results indicate that APA-mediated 3′ UTR lengthening is involved in gene expression regulation in multiple cellular senescence systems.

### Genes preferred distal pA sites in senescent cells enrich in senescence-associated pathways

To further understand the correlation between pA site selection and cellular senescence, we performed pathway analysis on genes preferring distal pA sites in PD11 compared with PD6 MEFs. Genes containing a significantly longer 3′ UTR in PD11 compared with PD6 MEFs were enriched in pathways that are highly pertinent to cellular senescence (Fig. 1F), as were the genes whose 3′ UTRs progressively lengthened in four different passages of MEFs (PD6, PD8, PD10, and PD11) (Supplemental Fig. S12). We also found similar enrichment in senescence-associated pathways between old and young rVSMCs (Fig. 1G). Notably, four of the shared pathways in senescent MEFs and rVSMCs, including ubiquitin-mediated proteolysis, the Wnt signaling pathway, cell cycle, and regulation of the actin cytoskeleton (Fig. 1F,G), are linked to cellular senescence (Amberg et al. 2012; Chandler and Peters 2013; Deschenes-Simard et al. 2014; Hofmann et al. 2014). Further examination of the genes preferring distal pA sites in mouse and rat revealed that these two species also possessed common genes undergoing APA regulation (Fig. 1H), which may serve as good candidates to study the function of 3′ UTR lengthening in cellular senescence.

### APA-induced longer 3′ UTR of *Rras2* reduces protein production and promotes cellular senescence

**Figure 2.**
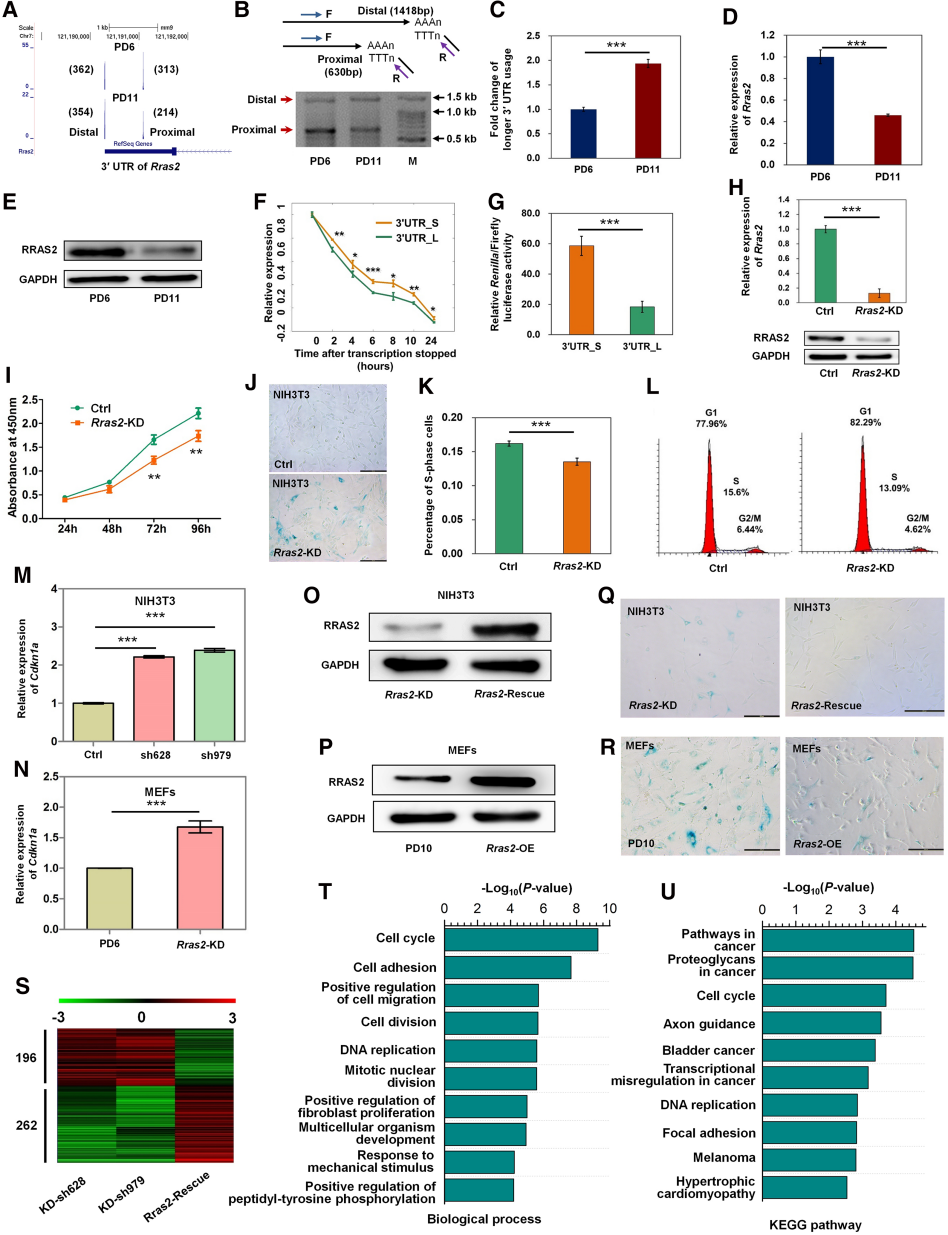
Down-regulation of *Rras2* through 3′ UTR lengthening promotes cellular senescence in mouse cells. (*A*) PA-seq track of proximal and distal pA site usage in PD6 and PD11 MEFs. Raw tag numbers reflected by both width and height of each pA peak are indicated in parentheses. (*B*) Changes of distal and proximal pA site usage between PD6 and PD11 MEF cells detected by 3′ RACE. The 3′ RACE strategy is shown in the *upper* panel. F and R indicate forward and reverse primers, respectively. PCR product sizes for distal and proximal pA sites are shown in parentheses. (*C*) The *Rras2* gene has increased usage of the distal pA site in MEF PD11 compared to PD6 detected by qRT-PCR. (*D*,*E*) qRT-PCR (*D*) and Western blot (*E*) of *Rras2* expression in PD6 and PD11 MEFs. *Gapdh* serves as the internal control. (*F*) Stability comparison of *Rras2* isoforms with 3′ UTR_S and 3′ UTR_L in mouse fibroblast cell line NIH3T3. (*G*) Luciferase activity from a reporter system containing 3′ UTR_S or 3′ UTR_L. (*H*) qRT-PCR (*upper* panel) and Western blot (*lower* panel) validation of *Rras2* knockdown in NIH3T3 cells. (*I*) Proliferation rate evaluation of NIH3T3 cells without (Ctrl) and with *Rras2* knockdown (*Rras2*-KD, sh979) by Cell Counting Kit-8 (CCK-8). (*J*) SA-β-gal staining in NIH3T3 cells without (Ctrl) and with *Rras2* knockdown (*Rras2*-KD). (*K*) FACS analysis of four replicate samples showed a lower percentage of *Rras2*-KD NIH3T3 cells in S phase compared with control cells. (*L*) Percentage of cells in S, G1, and G2/M phase in one replicate. (*M*,*N*) *Rras2* depletion promoted *Cdkn1a* expression in mouse NIH3T3 cells (*M*) and MEFs (*N*) evaluated by qRT-PCR. (*O*,*P*) Western blot validations of *Rras2* rescue in *Rras2*-KD NIH3T3 cells (*O*) and *Rras2*-overexpression (OE) in senescent MEFs (*P*). GAPDH serves as the internal control. (*Q*) Higher SA-β-gal staining caused by reduced *Rras2* expression was rescued by overexpression of *Rras2* (*Rras2*-OE) in mouse NIH3T3 cells. (*R*) *Rras2*-OE reversed senescence in primary MEF cells as evaluated by SA-β-gal staining assay. (*S*) Heat map of log_2_-transformed expression ratio of differentially expressed genes shared by *Rras2*-KD and *Rras2*-Rescue cells compared with the control NIH3T3 cells. Red and green denotes increased and decreased expression, respectively. The numbers of down- and up-regulated genes are shown on the *left*. (*T*,*U*) Enrichment of all differentially expressed genes shown in *S* for biological process (*T*) and the KEGG pathway (*U*), as determined by DAVID. (***) *P* < 0.001, (**) *P* < 0.01, (*) *P* < 0.05, two-tailed *t*-test.

To identify candidate genes that can affect cellular senescence through alternate pA site usage, we applied the following criteria: (1) favoring distal pA site usage both in senescent mouse and rat cells; (2) belonging to the shared four senescence-associated pathways (Fig. 1H); and (3) exhibiting decreased expression during senescence. Based on these criteria, we focused on *Rras2*. The tendency of *Rras2* to use distal pA sites in senescent cells was further confirmed. The UCSC Genome Browser displayed a higher usage of distal pA tags in both senescent MEFs (Fig. 2A) and rVSMCs (Supplemental Fig. S13). 3′ Rapid Amplification of cDNA Ends (3′ RACE) showed a reduced amplicon intensity of the proximal pA site in PD11 MEFs compared with PD6 MEFs (Fig. 2B). Real-time reverse transcription polymerase chain reaction (qRT-PCR) demonstrated a higher usage of longer 3′ UTRs in senescent MEFs (Fig. 2C). *Rras2* mRNA and protein levels were both down-regulated in senescent MEFs (Fig. 2D,E), consistent with the idea that longer 3′ UTRs tended to display a decreased mRNA abundance. To investigate how APA-induced increase in *Rras2* 3′ UTR length contributes to gene expression changes, we evaluated the RNA degradation rate of *Rras2* transcripts containing long and short 3′ UTRs. After blocking transcription followed by qRT-PCR, we found that *Rras2* transcripts with a longer 3′ UTR were less stable than those with a shorter 3′ UTR (Fig. 2F). We then inserted the shorter 3′ UTR (represented as 3′ UTR_S) and the longer 3′ UTR (3′ UTR_L) with a mutated proximal PAS of *Rras2* into a dual-luciferase reporter system, respectively. The reporter gene containing 3′ UTR_L exhibited significantly reduced luciferase activities compared with that containing 3′ UTR_S (Fig. 2G), implying that the APA-induced longer 3′ UTR of *Rras2* down-regulated RRAS2 protein abundance.

To determine whether decreased levels of RRAS2 trigger senescence-associated phenotypes, we depleted RRAS2 with two short hairpin RNAs (shRNA-mediated knockdown; *Rras2*-KD) in mouse NIH3T3 cells (Fig. 2H; Supplemental Fig. S14A) and observed delayed cell proliferation and increased SA-β-gal staining (Fig. 2I,J; Supplemental Fig. S14B,C). Cell cycle analysis showed that RRAS2-depletion reduced the percentage of S phase cells (Fig. 2K,L). *Cdkn1a*, which encodes cyclin-dependent kinase inhibitor 1A (P21), showed increased expression upon *Rras2-*KD (Fig. 2M). Moreover, *Rras2*-KD in primary MEFs has similar effects to those in NIH3T3 cells (Fig. 2N; Supplemental Fig. S15). In addition, reintroduction of RRAS2 (*Rras2*-Rescue) into *Rras2-*KD cells rescued the expression level of RRAS2 (Fig. 2O) and gave rise to reduced SA-β-gal signals (Fig. 2Q). Through RNA-seq profiling of *Rras2*-KD and *Rras2*-Rescue NIH3T3 cells, we found that altered RRAS2 expression caused expression changes in a variety of genes (Fig. 2S). Gene Ontology (GO) and pathway analysis revealed that those genes showing opposite expression trends were enriched in senescence-relevant biological processes, including cell cycle, cell adhesion, and DNA replication (Fig. 2T,U; Supplemental Table S5). More importantly, overexpression of RRAS2 (Fig. 2P) was able to reverse the SA-β-gal staining (Fig. 2R) and rescue *Cdkn1a* expression levels in senescent MEFs (Supplemental Fig. S16). Taken together, these data indicate that RRAS2 plays a crucial role in delaying cellular senescence.

**Figure 3.**
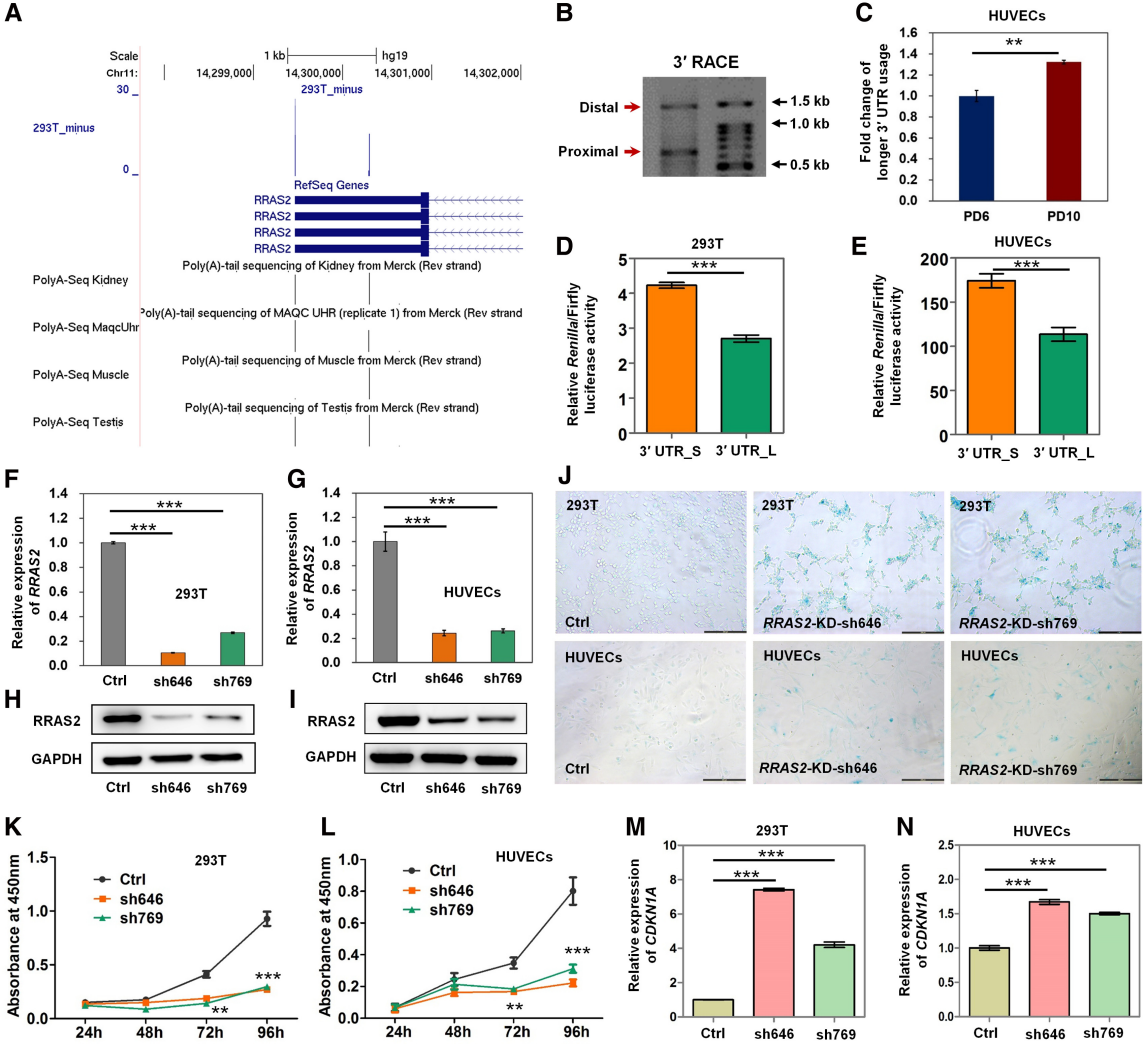
Decreased RRAS2 protein level via 3′ UTR lengthening causes senescence in human cells. (*A*) PA-seq track of 293T cells and PolyA-seq track of four representative human samples for *RRAS2* in the UCSC Genome Browser. (*B*) 3′ RACE products separated by agarose gel electrophoresis to confirm distal and proximal pA sites in human cells. *Right* lane, molecular weight marker. (*C*) *RRAS2* has a higher usage of the distal pA site in HUVECs at PD10 than at PD6, as determined by qRT-PCR. (*D*,*E*) Luciferase activity from a reporter containing the short 3′ UTR compared with that from the reporter containing 3′ UTR_L in 293T cells (*D*) and HUVECs (*E*). (*F*,*G*) Validation of *RRAS2* knockdown by two different shRNAs (sh646 and sh769) by qRT-PCR in 293T cells (*F*) and HUVECs (*G*). (*H*,*I*) Western blot confirmation of RRAS2 knockdown in 293T cells (*H*) and HUVECs (*I*). GAPDH served as the loading control. (*J*) SA-β-gal staining for 293T cells (*upper* panels) and HUVECs (*lower* panels) without (Ctrl) and with *RRAS2* knockdown (*RRAS2*-KD). Scale bar, 200 µm. (*K*,*L*) CCK-8 analysis of 293T cell (*K*) and HUVEC (*L*) proliferation without (Ctrl) and with *RRAS2* knockdown. (*M*,*N*) Increased *CDKN1A* expression upon *RRAS2* knockdown in 293T cells (*M*) and HUVECs (*N*), as determined by qRT-PCR. (***) *P* < 0.001, (**) *P* < 0.01, two-tailed *t*-test.

To address whether APA in *Rras2* is evolutionarily conserved between rodents and human, we verified the existence of its two pA sites in both human embryonic kidney (HEK) 293T cells and human umbilical vein endothelial cells (HUVECs) (Kim et al. 2007; Muck et al. 2008; Cardus et al. 2013; Zhang et al. 2014). Our PA-seq data from 293T cells (Ni et al. 2013) and public PolyA-seq data from human tissues confirmed the existence of both pA sites in human *RRAS2* (Fig. 3A). The two pA sites were validated by 3′ RACE (Fig. 3B), and their PCR products were cloned and sequenced by the Sanger method to confirm the existence of poly(A) or poly(T) sequence at the end of the amplicon (Supplemental Fig. S17). Of note, senescent HUVEC cells also favored the distal pA site (Fig. 3C), which generated less protein (Fig. 3D,E). Knockdown of RRAS2 (Fig. 3F–I) increased SA-β-gal activity (Fig. 3J), slowed cell proliferation rate (Fig. 3K,L), and induced *CDKN1A* expression in both 293T cells and HUVECs (Fig. 3M,N). These findings indicate an evolutionarily conserved mechanism that longer 3′ UTR of *Rras2* causes reduced protein production and then results in senescence in both rodents and human cells.

### Splicing factor TRA2B represses RRAS2 protein level through binding to its alternative 3′ UTR and contributes to cellular senescence

**Figure 4.**
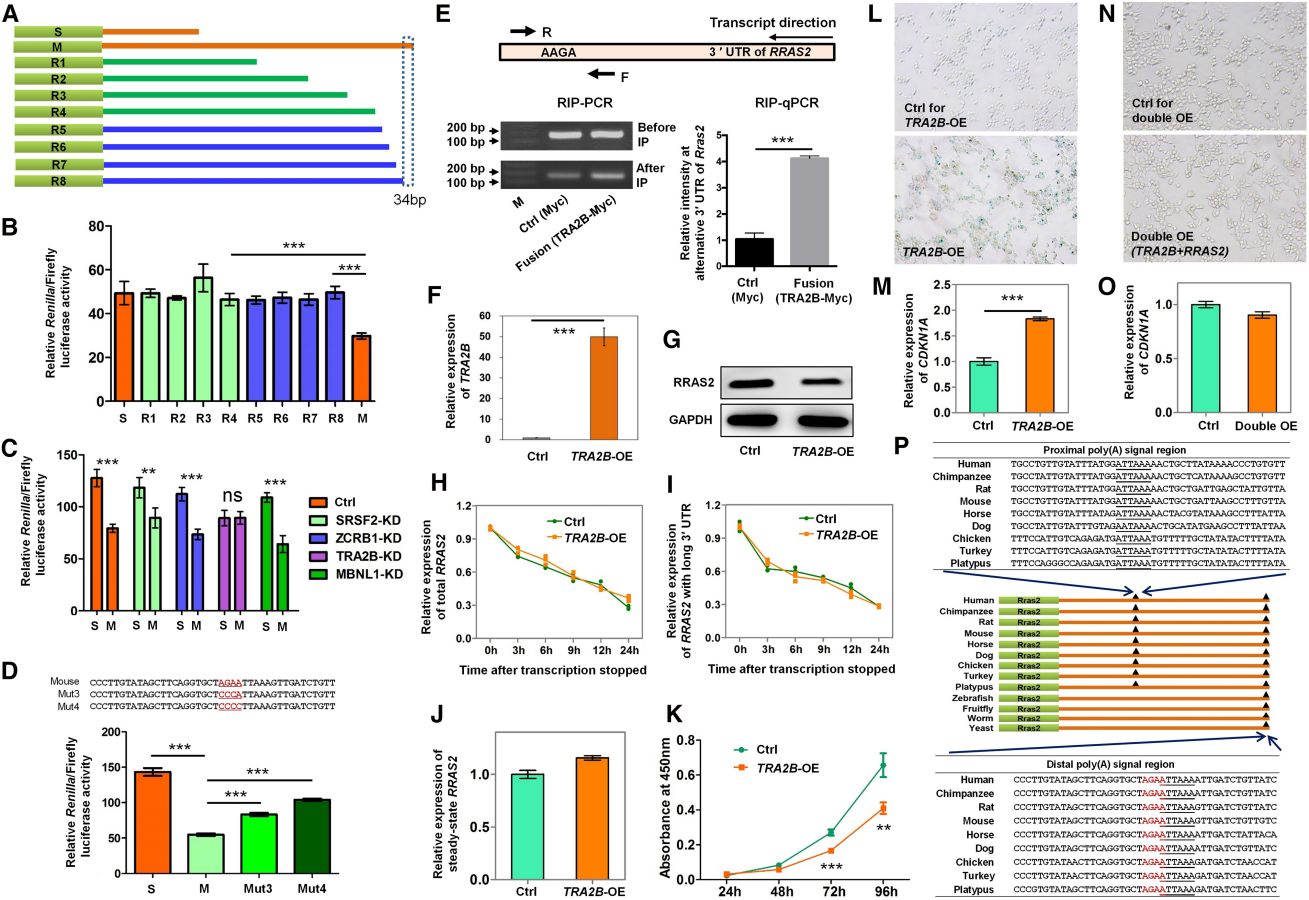
Binding of TRA2B to the alternative 3′ UTR of *Rras2* causes cellular senescence via reduced RRAS2 expression. (*A*) Schematic diagram of the truncation strategy to identify key *cis* regulatory elements. M denotes full length 3′ UTR with mutated proximal poly(A) signal. S represents 3′ UTR using the proximal pA site. R1–R4 refer to four truncated 3′ UTR fragments constructed for the first round screen. R5–R8 denote four additional constructs for the second-round screen. (*B*) Luciferase assays for all constructs indicated in *A*. (*C*) Luciferase assay screen for key *trans*-acting factors in candidate RBP knockdown cells. (ns) Not significant. (*D*) Luciferase assay in mouse cells transfected with constructs containing mutations introduced in the potential region containing the key *cis*-element. “AGAA” is the core binding motif of TRA2B (Grellscheid et al. 2011). (*E*) TRA2B binding to the alternative 3′ UTR of *Rras2* evaluated by RIP-PCR and RIP-qPCR. The PCR primer pair (F and R, forward and reverse, respectively) was designed to span the “AGAA” motif. TRA2B was fused to Myc protein to serve as the ectopically expressed fusion protein. A vector expressing Myc protein served as the internal control (Ctrl). Anti-Myc was used to pull down RNA from MEF cells either expressing Ctrl (Myc) or Fusion (TRA2B-Myc) protein. The same amount of RNA was applied for RIP-PCR (*left* panel) and RIP-qPCR (*right* panel). (*F*) qRT-PCR validation of *TRA2B* overexpression (*TRA2B*-OE) in human 293T cells. (*G*) Decreased RRAS2 protein level upon *TRA2B* overexpression detected by Western blot in 293T cells. (*H*,*I*) *RRAS2* RNA degradation rate was assayed for total isoforms (*H*) and isoform with longer 3′ UTRs (*I*) in *TRA2B*-OE and control human cells by qRT-PCR. (*J*) Steady-state mRNA level of *RRAS2* was quantified in *TRA2B*-OE and control human cells by qRT-PCR. (*K*) Overexpression of *TRA2B* slows down cell proliferation as detected by the CCK-8 assay. (*L*,*M*) *TRA2B*-OE caused increased SA-β-gal staining (*L*) and *CDKN1A* expression (*M*) in 293T cells. (*N*,*O*) Overexpression of *RRAS2* in TRA2B up-regulated human cells reversed SA-β-gal staining (*N*) and *CDKN1A* expression (*O*). (*P*) Sequence alignment near proximal and distal pA sites of *Rras2* in representative species. The PAS is underlined while the TRA2B core binding motif “AGAA” is highlighted in red text. (***) *P* < 0.001, (**) *P* < 0.01, two-tailed *t*-test.

We reasoned that *cis*-elements recognized by either miRNAs or RBPs in the alternative 3′ UTR of *Rras2* contribute to decreased RRAS2 protein production. To this end, we divided the 3′ UTR of *Rras2* into four regions by deleting sequences of different lengths (shown as R1–R4 in Fig. 4A) and inserted them separately into a luciferase reporter, which was then transfected into mouse cells. Subsequent luciferase assays demonstrated significantly reduced luciferase activity from the construct containing a long 3′ UTR (resulting from mutated proximal PAS, labeled “M”) compared with the short 3′ UTR (labeled “S”) and the R1–R4 constructs (Fig. 4B). Thus, the sequence of last 148 base pairs (bp) located in the alternative 3′ UTR of *Rras2* contains key motifs or elements essential for protein production.

To further narrow the range of key elements, four more reporter constructs harboring the truncated 3′ UTRs (presented as R5–R8 in Fig. 4A) were generated. The construct lacking a 34-bp element showed significantly reduced luciferase activity, indicating that this element is critical for down-regulation of protein production (Fig. 4A,B). No miRNA binding sites were predicted in this 34-bp region; therefore, we searched RBPmap (Paz et al. 2014) for potential RBPs that might recognize this element and identified four RBPs (SPSF2, ZCRB1, TRA2B, and MBNL1). Following individual knockdown of the four RBPs, we measured the luciferase activities of S and M constructs (Fig. 4C). Depletion of TRA2B, a known splicing factor involved in mRNA processing, cell proliferation, and migration (Yang et al. 2015), eliminated the difference in luciferase activities between the S and M fragments-containing reporters (Fig. 4C), suggesting that TRA2B played an important role in down-regulating protein production. These data implied that the 34-bp element in the 3′ UTR of *Rras2* is required for TRA2B-mediated down-regulation of RRAS2.

The “AGAA” element may serve as the core sequence of the TRA2B binding motif (Fig. 4D; Grellscheid et al. 2011). Considering that “AGAA” shared 1 nt with the downstream PAS “ATTAAA” in the alternative 3′ UTR of *Rras2*, we mutated the first 3 nt to avoid disruption of the PAS (“AGAA” to “CCCA”) (designated Mut3 in Fig. 4D). All 4 nt were also mutated (“AGAA” to “CCCC”) (denoted Mut4 in Fig. 4D) to validate the effects that are dependent on the whole core sequence. According to luciferase reporter assays, the Mut3 construct partially restored luciferase activity compared with that for M, while Mut4 showed a higher capability of restoring the repressed luciferase activity than Mut3 (Fig. 4D). To explore whether TRA2B directly binds to the alternative 3′ UTR of *Rras2*, we carried out RNA immunoprecipitation coupled with both regular and quantitative PCR (RIP-PCR and RIP-qPCR) assays. The results showed an enrichment of TRA2B-binding signal (Fig. 4E), suggesting direct binding of TRA2B to the alternative 3′ UTR of *Rras2*. To further evaluate the role of “AGAA,” we moved this motif to two upstream positions in the alternative 3′ UTR of *Rras2*. The “AGAA” motif at a more proximal position did not repress protein production (Supplemental Fig. S18), implying that the “AGAA” motif in the alternative 3′ UTR of the *Rras2* gene was required but not sufficient to repress protein levels. Together, these data suggest that the “AGAA” motif within the alternative 3′ UTR of *Rras2* is essential for TRA2B binding and repression of RRAS2 protein production.

To further confirm the contribution of TRA2B-RRAS2 regulation to cellular senescence, we overexpressed *TRA2B* in 293T cells, which show a higher usage of the distal pA site in *RRAS2* and can serve as an appropriate cell model to perform the test, given that TRA2B bound to the alternative 3′ UTR of *Rras2* (Fig. 3A). As expected, ectopic expression of TRA2B led to decreased RRAS2 protein level (Fig. 4F,G). Notably, neither RNA stability (Fig. 4H,I) nor mRNA steady-state levels of *RRAS2* (Fig. 4J) showed considerable changes upon TRA2B overexpression, suggesting that the reduced RRAS2 protein level was caused by repressed translation through binding of TRA2B to the alternative 3′ UTR*.* Up-regulation of TRA2B led to a reduced proliferation rate (Fig. 4K), higher SA-β-gal staining level, and increased *CDKN1A* expression (Fig. 4L,M). Recovery of RRAS2 expression by additional overexpression of RRAS2 in TRA2B-overexpressed cells attenuated the *CDKN1A* expression and SA-β-gal staining to basal levels (Fig. 4N,O). Taken together, these data demonstrated that binding of TRA2B to the alternative 3′ UTR of *Rras2* results in decreased RRAS2 expression, which in turn accelerated cellular senescence.

### Key elements involved in APA regulation of *Rras2* are evolutionarily conserved

Since *Rras2* prefers the distal pA site in senescent mouse, rat, and human cells (Figs. 2A–C, 3C; Supplemental Fig. S13), it would be interesting to have an evolutionary view on related *Rras2* sequences. Comparative genomic analysis revealed that the 3′ UTR of *Rras2* was extremely conserved in representative species from galliformes to primates (Supplemental Fig. S19), implying that regulation of 3′ UTR length by APA is of evolutionary significance. Notably, all listed animals contained a canonical PAS (AA/TTAAA) near the proximal pA site (Fig. 4P). This strongly indicated that APA-regulated *Rras2* expression plays a crucial role during evolution. The sequence adjacent to the distal pA site within the 3′ UTR of *Rras2* also contained the canonical PAS “ATTAAA” and the TRA2B binding motif “AGAA,” which were conserved from birds to mammals (Fig. 4P), indicating that a strong selection pressure is likely to have driven the molecular evolution for TRA2B-mediated regulation of APA in *Rras2*.

## Discussion

**Figure 5.**
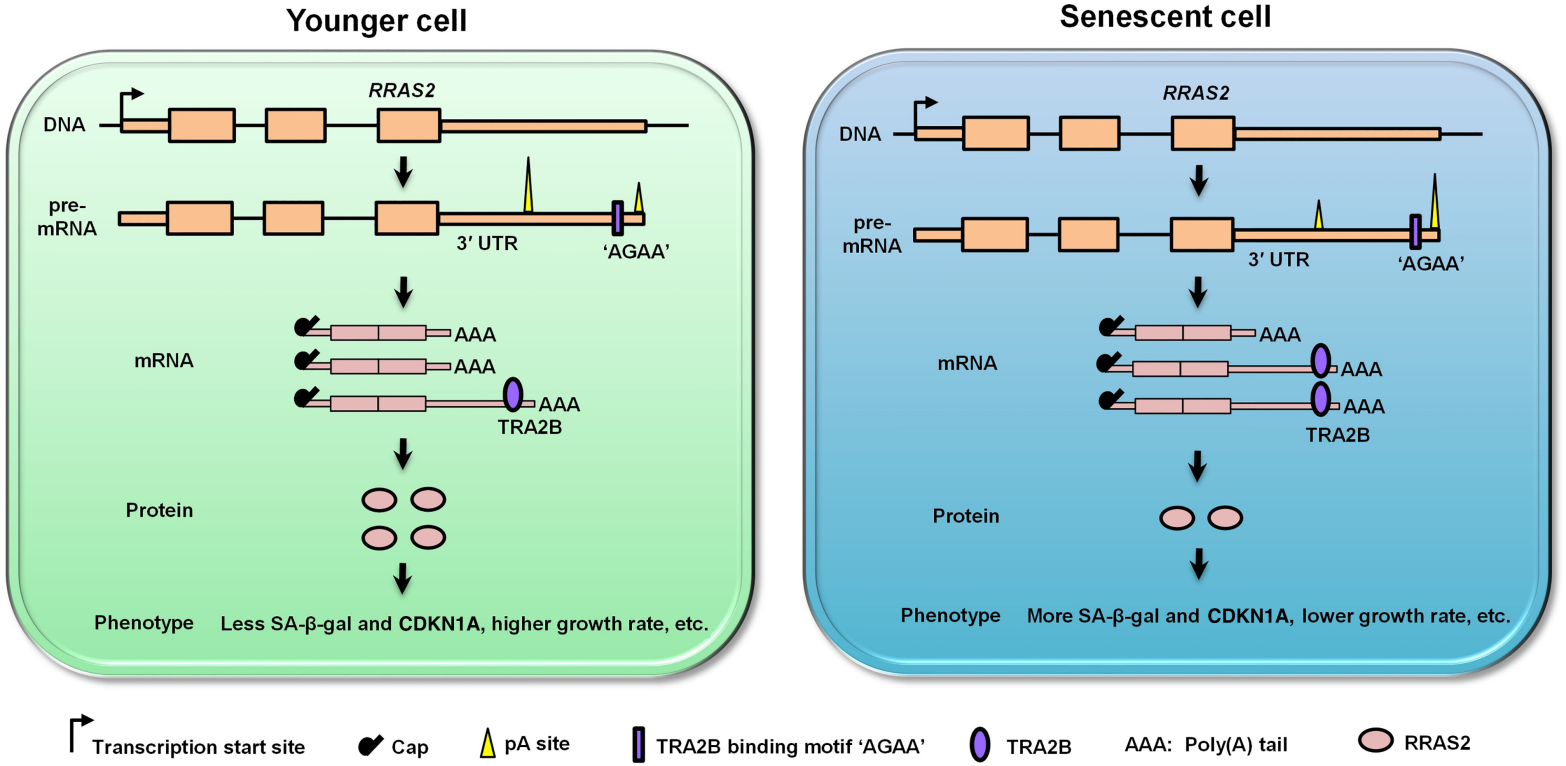
Working model for APA as a new mechanism in regulating cellular senescence. Genes, such as *RRAS2*, favor distal pA site usage during senescence, leading to 3′ UTR lengthening. Splicing factor TRA2B bound to its core *cis*-element “AGAA” located in the alternative 3′ UTR of *RRAS2* and repressed RRAS2 protein production, thereby leading to senescence-associated phenotypes.

In this study, we discovered that APA-mediated 3′ UTR lengthening played a role in cellular senescence. As exemplified by *Rras2* (Fig. 5), we extended the functional importance of APA to the aging field and provided a novel perspective for understanding the mechanism underlying cellular senescence. However, upstream factors controlling 3′ UTR lengthening during cellular senescence need to be further explored. Given that certain core factors in the 3′ processing machinery were known to play a role in APA regulation (Takagaki et al. 1996; Zheng and Tian 2014), we therefore surveyed the gene expression of 24 important polyadenylation *trans*-factors. Most of these factors underwent a trend of decreased expression during MEFs senescence (Supplemental Fig. S20), consistent with recent findings that up-regulation of polyadenylation factors was associated with 3′ UTR shortening (Mayr and Bartel 2009; Xia et al. 2014). These results provided a possible mechanistic explanation for 3′ UTR lengthening during cellular senescence and deserved further investigation.

Genes preferring distal pA sites showed a global lengthening of the 3′ UTR and a trend of decreased mRNA levels in both senescent MEFs and rVSMCs (Fig. 1), supplementing the observation that genes favoring proximal pA sites tended to have increased mRNA levels in cancer cells (Mayr and Bartel 2009; Xia et al. 2014). We also found that many genes displayed opposite pA site usage preference in senescent cells compared with cancer cells. Eighty-two and 166 genes that favored proximal pA sites in seven tumor types were prone to use distal pA sites in senescent MEFs and rVSMCs, respectively (Supplemental Fig. S21). In addition, 35 genes preferring shorter 3′ UTRs in multiple cancer cells tended to use longer 3′ UTRs in both senescent MEFs and rVSMCs (Supplemental Fig. S21). These findings supported a model that interaction between condition-specific *trans-*acting factors and dynamic changes in 3′ UTR length determined by APA could contribute to opposite biological processes, such as cellular senescence and tumor development.

There are two major categories of cellular senescence, developmentally programmed senescence and stress-induced premature senescence (SIPS) (Munoz-Espin and Serrano 2014). Here, we showed that MEFs in replicative senescence underwent global 3′ UTR lengthening. VSMCs derived from old rats were likely to undergo a combination of replicative senescence and varieties of stress-induced senescence. Whether SIPS itself will induce global 3′ UTR lengthening needs to be further determined. Thus, more senescence models are required to fully understand the prevalence and functional relevance of 3′ UTR lengthening. In conclusion, our results provide evidence that APA contributes to the regulation of gene expression during cell senescence in multiple species, implying APA-regulated gene expression may be evolutionarily conserved.

## Methods

### Cell isolation, cultivation, and total RNA extraction

Primary MEFs were isolated from embryos of a 12.5- to 14-d pregnant C57BL/6 mouse according to a previously described method (Todaro and Green 1963). Details were described in Supplemental Methods. Total RNA was isolated using TRIzol reagent (Invitrogen for both MEFs and rVSMCs from rats of different ages (2 wk and 2 yr).

### SA-β-gal staining, cell proliferating rate assay, and cell cycle analysis

SA-β-gal activity (Dimri et al. 1995) was monitored in mouse (MEFs, NIH3T3) and human (293T, HUVECs) cells using a senescence detection kit (BioVision, cat. no. K320-250). Mouse (NIH3T3) and human (293T, HUVECs) cells with or without candidate gene knockdown were cultured and assayed for cell proliferation using a Cell Counting Kit-8 (CCK-8) according to the vendor's instructions (Dojindo). Details of SA-β-gal staining and cell cycle analysis are described in Supplemental Methods.

### PA-seq and RNA-seq library construction, mapping, and peak calling

The PA-seq libraries were constructed according to our established protocol (Ni et al. 2013). The dUTP-based strand-specific RNA-seq libraries were constructed by following a previously described protocol (Parkhomchuk et al. 2009). Both types of libraries were sequenced using an Illumina HiSeq2000 platform in a paired-end 2 × 101-bp manner. Processed raw data were aligned to the mouse genome (version mm9) or rat genome (version rn5) using TopHat2 (Kim et al. 2013a). Detailed peak calling of pA sites and expression analysis were included in Supplemental Methods. All called pA clusters in MEFs are listed in Supplemental Table S6.

### Comparison of APA profiles between MEFs of different passages

The tandem 3′ UTR isoform switch index values were calculated according to a published approach (Fu et al. 2011; Li et al. 2012). Genes with expression fold-change ≥ 1.5 and FPKM ≥ 1 in both samples are shown in Figure 1, D and E. The genes with a *P*-value cut-off of 0.05 (corresponding to a false discovery rate [FDR], estimated by the Benjamini–Hochberg method with R software [R Core Team 2015]) were considered to have significantly changed APA among the different passages. Specifically, an FDR ≤ 0.05 with a positive TSI (Li et al. 2012) implies a lengthening 3′ UTR across the different passages of cellular senescence; an FDR ≤ 0.05 with a negative TSI implies a shortening 3′ UTR. All APA genes with detailed information, including expression value, effective 3′ UTR length, and RUD index (Ji et al. 2011), are included in Supplemental Table S7. Comparison of lengthening or shortening of 3′ UTRs is included in Supplemental Table S8.

### Pathway enrichment analysis

The Database for Annotation, Visualization, and Integrated Discovery (DAVID) (Huang da et al. 2009) was used for the pathway enrichment analysis, and the Kyoto Encyclopedia of Genes and Genomes (KEGG) database was selected.

### Western blotting and qRT-PCR

Primary antibodies (RRAS2, 1:1000, cat. no. ab209078, Abcam; GAPDH, 1:2000–3000, cat. no. sc-32233, Santa Cruz Biotechnology; CDKN2A, 1:500–1000, cat. no. sc-1207, Santa Cruz Biotechnology) targeting proteins of interest were used for the Western blot. A detailed description is included in Supplemental Methods. Total RNA used for expression quantification was reverse-transcribed into cDNA using random primers, and then mRNA levels were quantified by qRT-PCR and normalized to that of *Gapdh* (Roche LightCycler). For pA site usage quantification, RNAs were reverse-transcribed with oligo(dT) primer, followed by PCR with two pairs of primers (proximal and distal) targeting different regions of the cDNAs. Specifically, the region targeted by the proximal pair is common to both APA isoforms and the region targeted by the distal pair is unique to the longer isoform. qPCR signals from the proximal and distal pairs of primers were compared to indicate the relative expression of the two isoforms. All primer sequence information is listed in Supplemental Table S9.

### RNA stability, RNA immunoprecipitation, and luciferase assay

For evaluation of RNA stability, cells were treated with actinomycin D (5 µg/mL) for 0, 2, 4, 6, 8, 10, and 24 h. After the indicated time, total RNAs were isolated and analyzed by qRT-PCR. Primer sequences are shown in Supplemental Table S9. RNA immunoprecipitation (RIP) was performed according to a previously published protocol with minor modification (Zhao et al. 2008). The Dual-Luciferase Reporter 1000 Assay System (Promega) was carried out to evaluate the impact of protein production for different lengths of 3′ UTRs. Detailed information of RIP-PCR and the luciferase assay are described in Supplemental Methods.

## Data access

The raw PA-seq and RNA-seq data from this study have been submitted to the NCBI Sequence Read Archive (SRA; https://www.ncbi.nlm.nih.gov/sra/) under accession number SRP065821.

## Supplementary Material

Supplemental Material
