# Supplementary material for: 3′ UTR lengthening as a novel mechanism in regulating cellular senescence
Source: Genome Res. 2018 Mar;28(3):285–94. doi: 10.1101/gr.224451.117 (PMC5848608; doi:10.1101/gr.224451.117)
Supplement: Supplemental Material [file supp_28_3_285__index.html]

3′ UTR lengthening as a novel mechanism in regulating cellular senescence — Supplemental Material 

# 3′ UTR lengthening as a novel mechanism in regulating cellular senescence

## Supplemental Material

- Supplemental\_Fig\_S1.docx
- Supplemental\_Fig\_S2.docx
- Supplemental\_Fig\_S3.docx
- Supplemental\_Fig\_S4.docx
- Supplemental\_Fig\_S5.docx
- Supplemental\_Fig\_S6.docx
- Supplemental\_Fig\_S7.docx
- Supplemental\_Fig\_S8.docx
- Supplemental\_Fig\_S9.docx
- Supplemental\_Fig\_S10.docx
- Supplemental\_Fig\_S11.docx
- Supplemental\_Fig\_S12.docx
- Supplemental\_Fig\_S13.docx
- Supplemental\_Fig\_S14.docx
- Supplemental\_Fig\_S15.docx
- Supplemental\_Fig\_S16.docx
- Supplemental\_Fig\_S17.docx
- Supplemental\_Fig\_S18.docx
- Supplemental\_Fig\_S19.docx
- Supplemental\_Fig\_S20.docx
- Supplemental\_Fig\_S21.docx
- Supplemental\_Table\_S1.docx
- Supplemental\_Table\_S2.docx
- Supplemental\_Table\_S3.docx
- Supplemental\_Table\_S4.docx
- Supplemental\_Table\_S5.xlsx
- Supplemental\_Table\_S6.xlsx
- Supplemental\_Table\_S7.xlsx
- Supplemental\_Table\_S8.xlsx
- Supplemental\_Table\_S9.xlsx
- Supplemental\_Methods.docx
