# Supplementary material for: 3′ UTR lengthening as a novel mechanism in regulating cellular senescence
Source: Genome Res. 2018 Mar;28(3):285–94. doi: 10.1101/gr.224451.117 (PMC5848608; doi:10.1101/gr.224451.117)
Supplement: Supplemental Material [file supp_gr.224451.117_Supplemental_Fig_S1.docx]

**
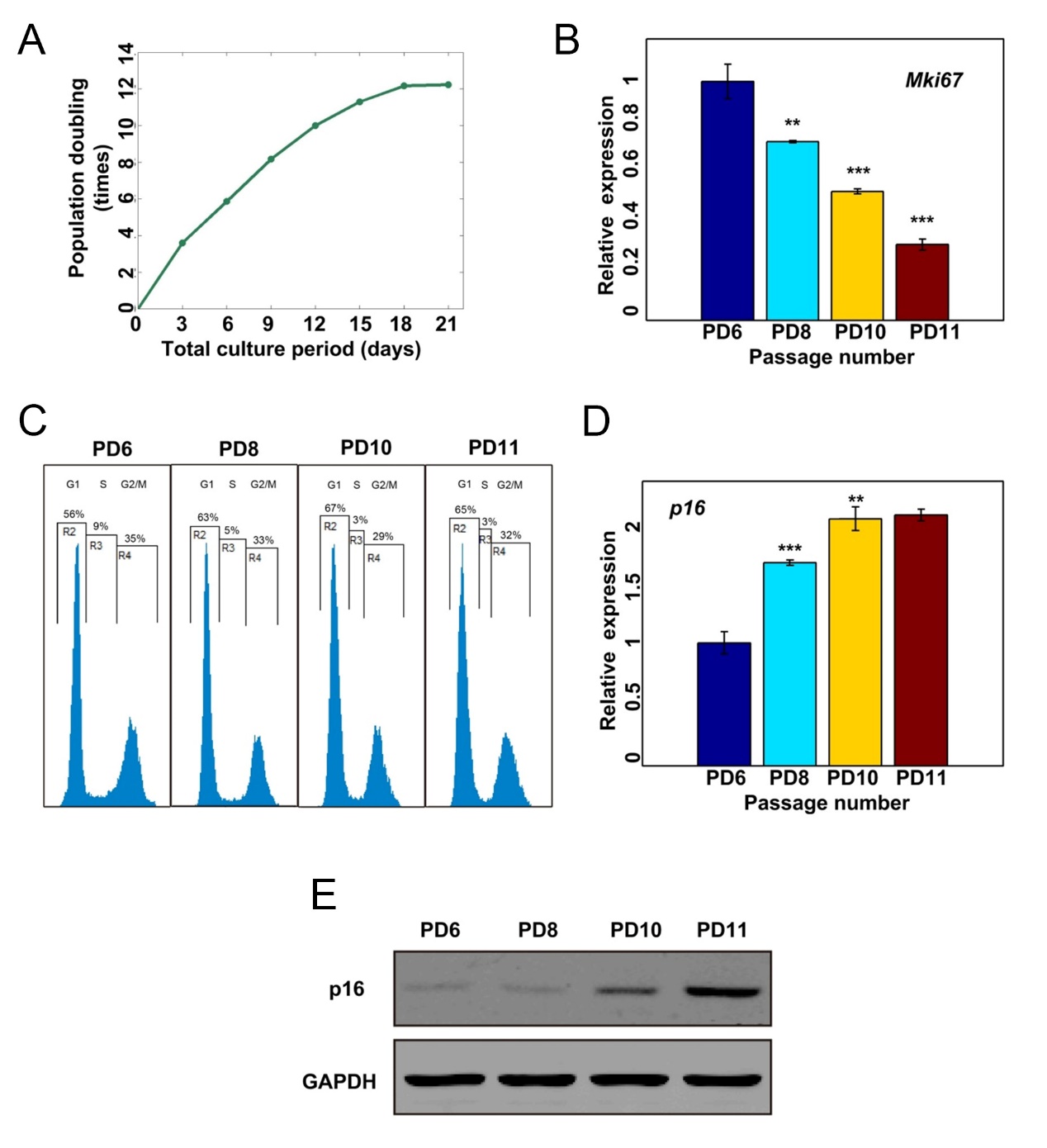
**

**Supplemental Figure S1. Establishment of a replicative senescence model for MEFs.** (A) MEFs were continuously sub-cultured and the population doubling number along culture time was recorded. (B) Relative expression level of *Mki67* in PD6, PD8, PD10, and PD11 MEF passages determined by qRT-PCR. (C) Fluorescence-activated cell sorting (FACS) analysis indicates a trend for an increased percentage of G1 phase cells and a decreased percentage of S phase cells among MEFs from PD6 to PD11. (D) Relative expression level of CDKN2A in PD6, PD8, PD10, and PD11 MEFs passages. (E) Relative abundance of CDKN2A protein in PD6, PD8, PD10, and PD11 MEFs passages determined by Western blot. (***) *P*$<$0.001 and (**) *P*$<$0.01, two tailed Student's *t*-test.
