# Supplementary material for: 3′ UTR lengthening as a novel mechanism in regulating cellular senescence
Source: Genome Res. 2018 Mar;28(3):285–94. doi: 10.1101/gr.224451.117 (PMC5848608; doi:10.1101/gr.224451.117)
Supplement: Supplemental Material [file supp_gr.224451.117_Supplemental_Fig_S2.docx]

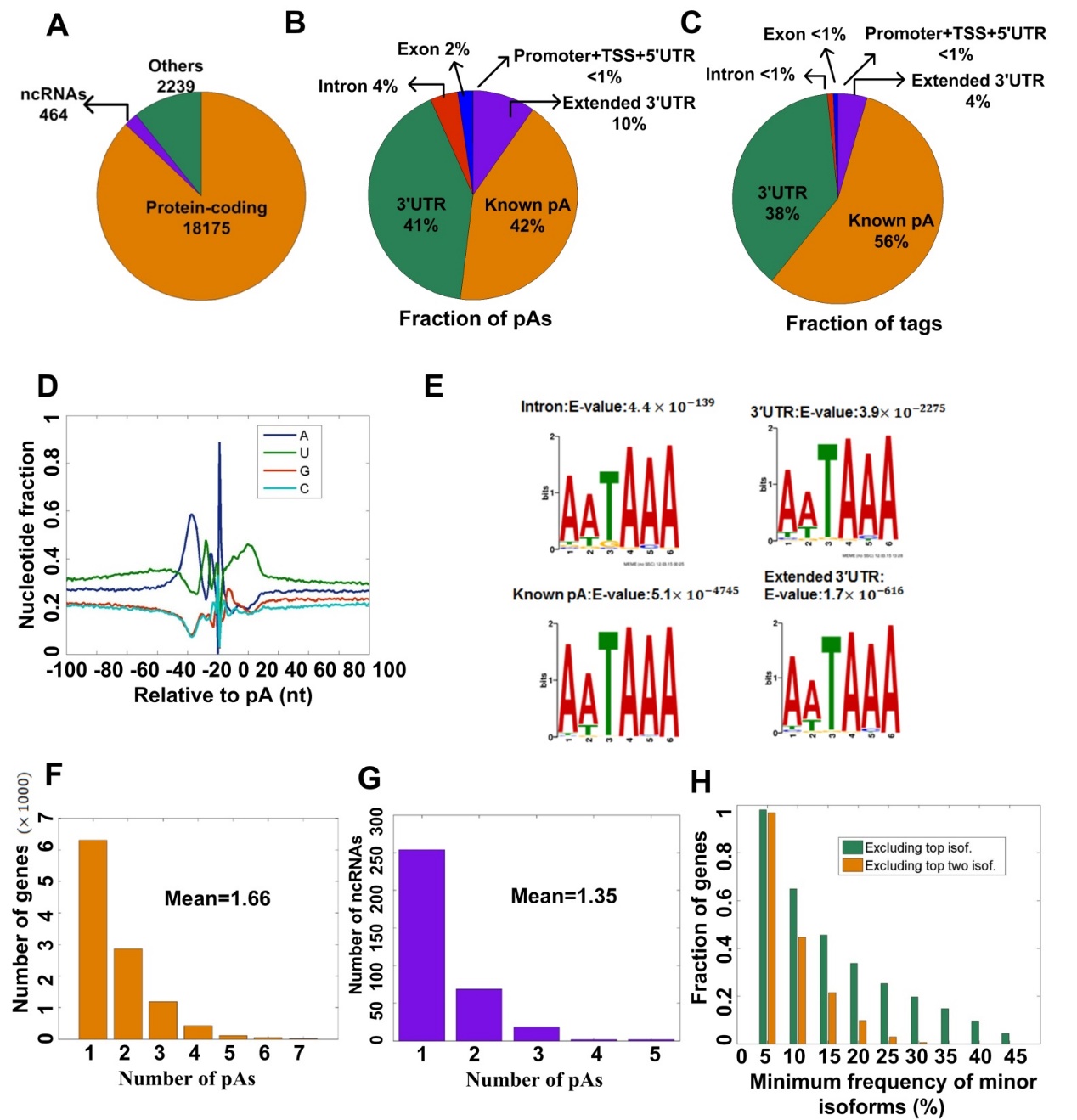


**Supplemental Figure S2. Summary of pAs in MEFs identified by PA-seq.** (A) Categories of genes with identified pAs based on RefSeq annotation. (B) Genomic location distribution of the identified pAs. (C) Genomic location distribution of tags in the identified pAs. (D) Distribution of nucleotide composition in the sequences around the identified pAs. (E) The canonical PAS motif, AATAAA, identified by analyzing the sequences from the upstream (-40 bp) of the pAs located in the intron, 3′ UTR, known pA, and extended 3′ UTR regions using MEME program, significant E-values were denoted. (F) Histogram of the number of protein-coding genes with different numbers of pAs. (G) Histogram of the number of non-coding genes with different numbers of pAs. (H) Fraction of genes with the minimum frequency of minor 3′ UTR isoforms exceeding given thresholds (0%, 5%, 15%, 20%, 25%, 30%, 35% 40%, and 45%). Frequencies of minor isoforms were calculated by excluding the top isoform (green bars) and the top two isoforms (orange bars).
