# Supplementary material for: 3′ UTR lengthening as a novel mechanism in regulating cellular senescence
Source: Genome Res. 2018 Mar;28(3):285–94. doi: 10.1101/gr.224451.117 (PMC5848608; doi:10.1101/gr.224451.117)
Supplement: Supplemental Material [file supp_gr.224451.117_Supplemental_Fig_S3.docx]

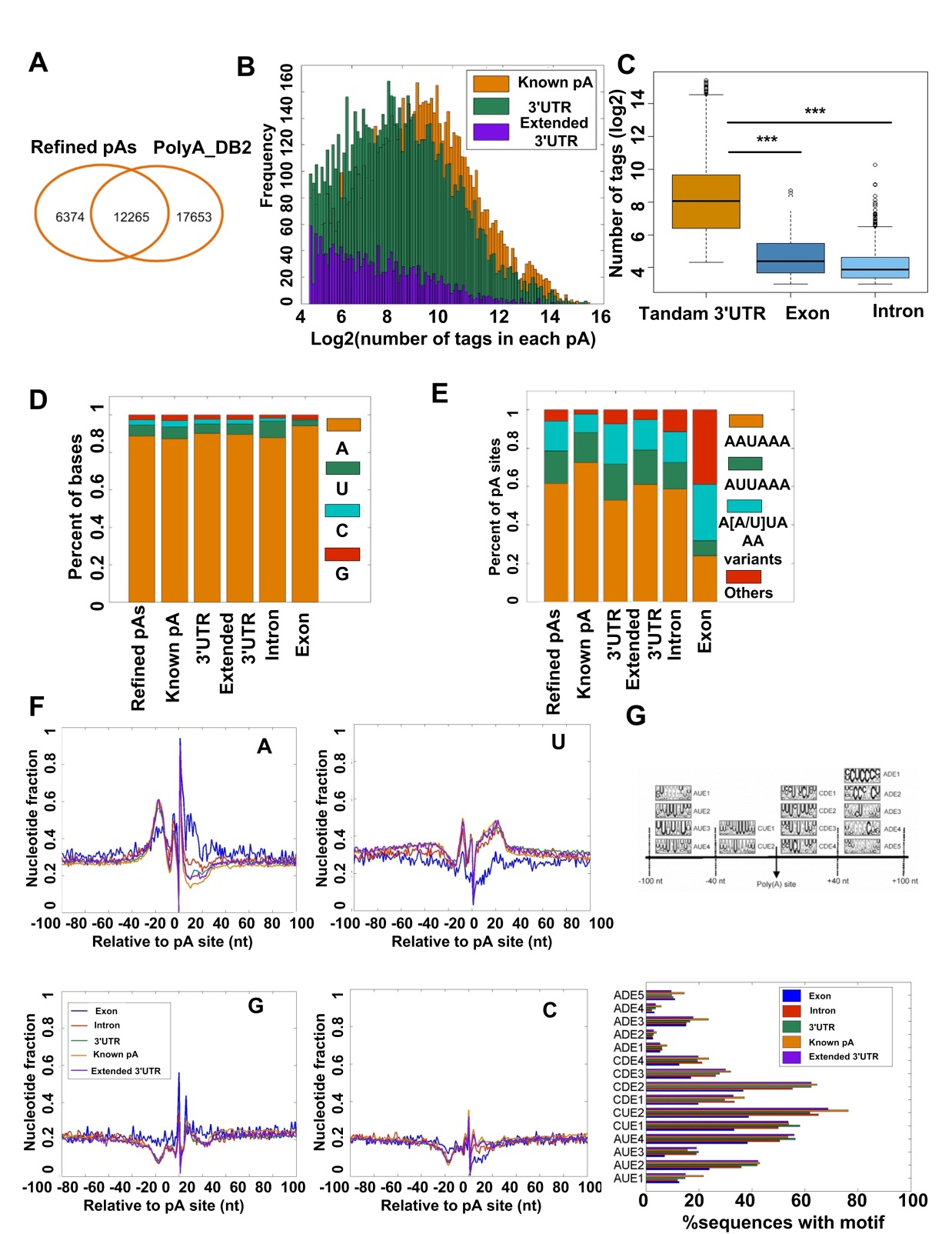


**Supplemental Figure S3. Base composition comparison of different types of pAs.** (A) Venn diagram between our identified pAs and those in the PolyA_DB2 database. (B) Distribution of the number of tags attributed to different types of tandem pAs. (C) Box plots for the number of tags attributed to pAs located in 3′ UTRs, exons and introns, respectively. (D) Nucleotide composition of the 3′ base next to pAs from different genomic locations. (E) Distribution of PAS sequences in the -40 to -1 nt region of pAs from different genomic locations. (F) Base distributions in the 200 nt surrounding pAs from different genomic locations. (G) Frequencies of matched motifs defined by Hu et al. (RNA, 2005) in the 200 nt surrounding pAs from different genomic locations were predicted by polya_svm. (***) *P*$<$0.001, two Mann-Whitney U test.
