# Supplementary material for: 3′ UTR lengthening as a novel mechanism in regulating cellular senescence
Source: Genome Res. 2018 Mar;28(3):285–94. doi: 10.1101/gr.224451.117 (PMC5848608; doi:10.1101/gr.224451.117)
Supplement: Supplemental Material [file supp_gr.224451.117_Supplemental_Fig_S4.docx]

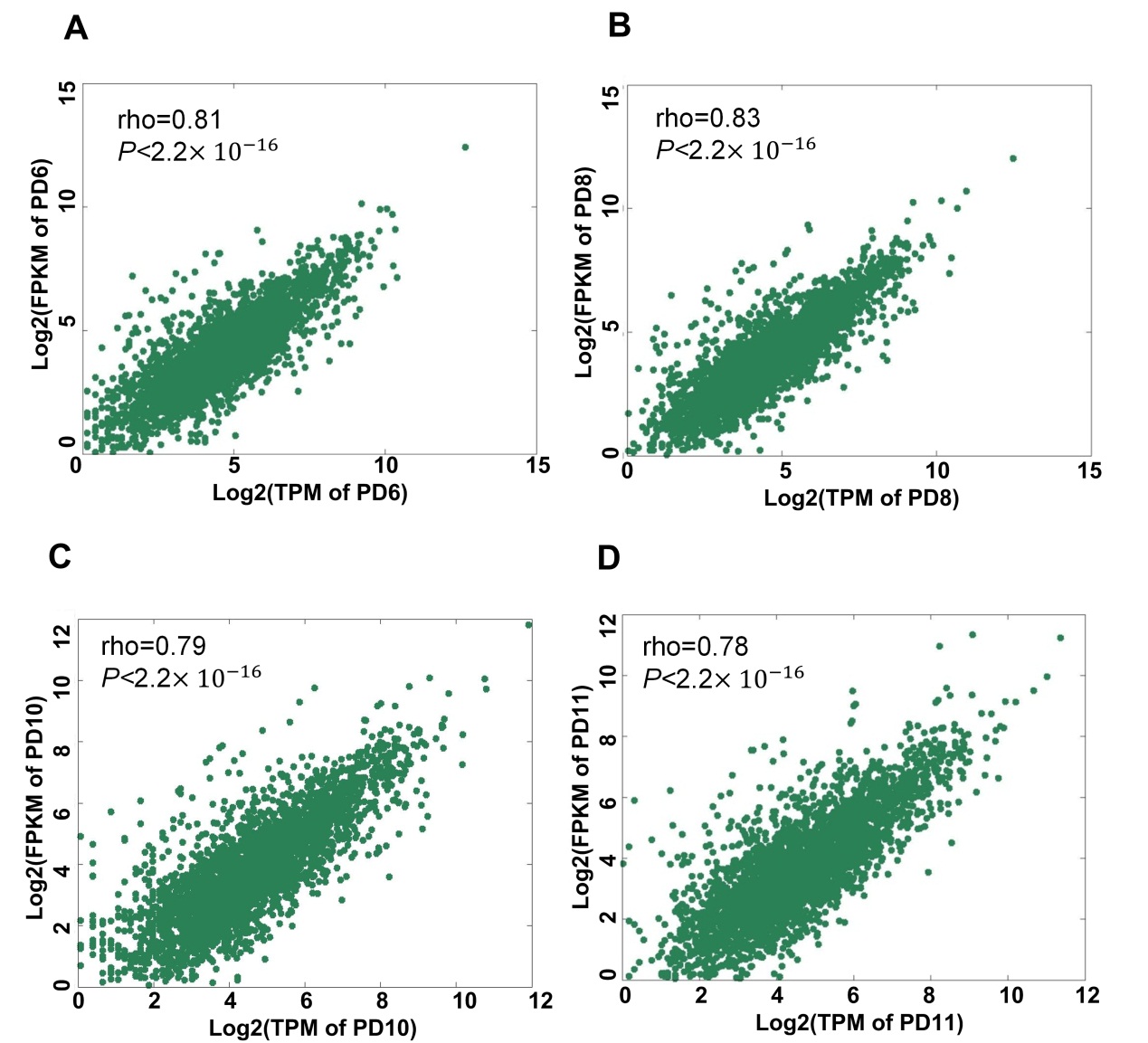


**Supplemental Figure S4. Correlation of expression measured by PA-seq and RNA-seq for genes with multiple pAs.** (A-D) Correlation of expression measured by PA-seq and RNA-seq for genes with APA regulation in PD6, PD8, PD10, and PD11. Rho indicates Spearman's rank correlation coefficient.
