# Supplementary material for: 3′ UTR lengthening as a novel mechanism in regulating cellular senescence
Source: Genome Res. 2018 Mar;28(3):285–94. doi: 10.1101/gr.224451.117 (PMC5848608; doi:10.1101/gr.224451.117)
Supplement: Supplemental Material [file supp_gr.224451.117_Supplemental_Fig_S5.docx]

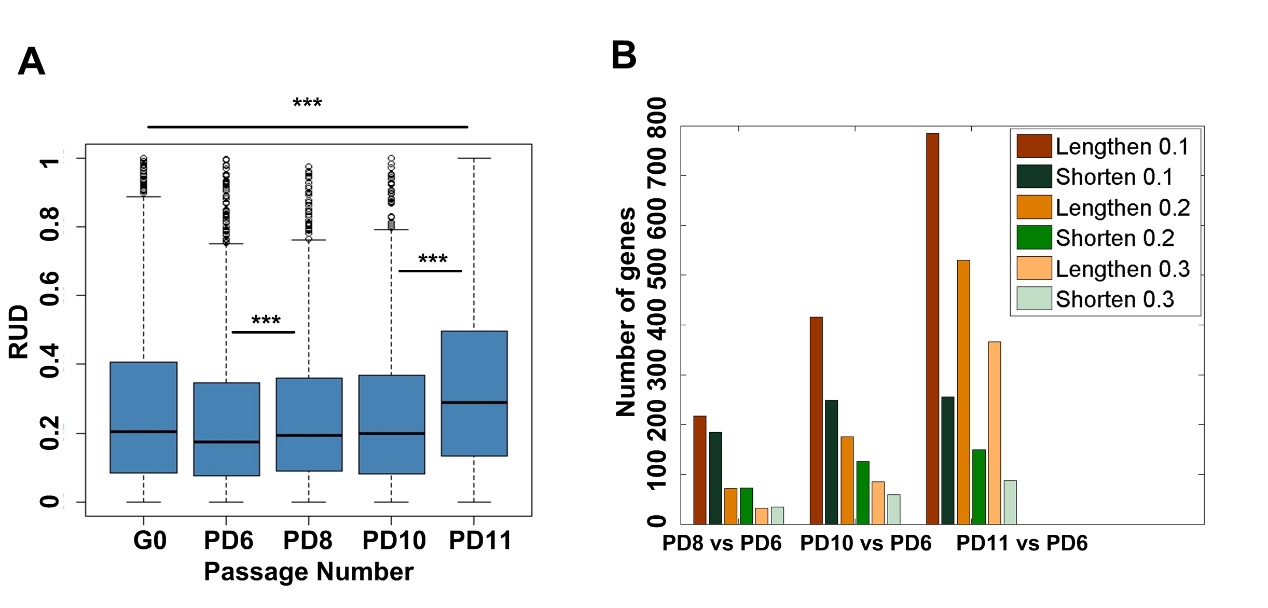


**Supplemental Figure S5. Global lengthening of 3′ UTRs for genes with APA regulation during replicative senescence of MEFs.** (A) Box plot for relative expression of mRNA isoforms using distal pA sites (RUD scores) across G0, PD6, PD8, PD10, and PD11 of MEFs. (B) Number of genes with higher RUD scores and number of genes with lower RUD scores by comparing PD11, PD10, and PD8 to PD6 given different cutoffs based on RNA-seq data. (***) P < 0.001 and (*) P < 0.05, two-tailed Wilcoxon signed rank test.
