# Supplementary material for: 3′ UTR lengthening as a novel mechanism in regulating cellular senescence
Source: Genome Res. 2018 Mar;28(3):285–94. doi: 10.1101/gr.224451.117 (PMC5848608; doi:10.1101/gr.224451.117)
Supplement: Supplemental Material [file supp_gr.224451.117_Supplemental_Fig_S6.docx]

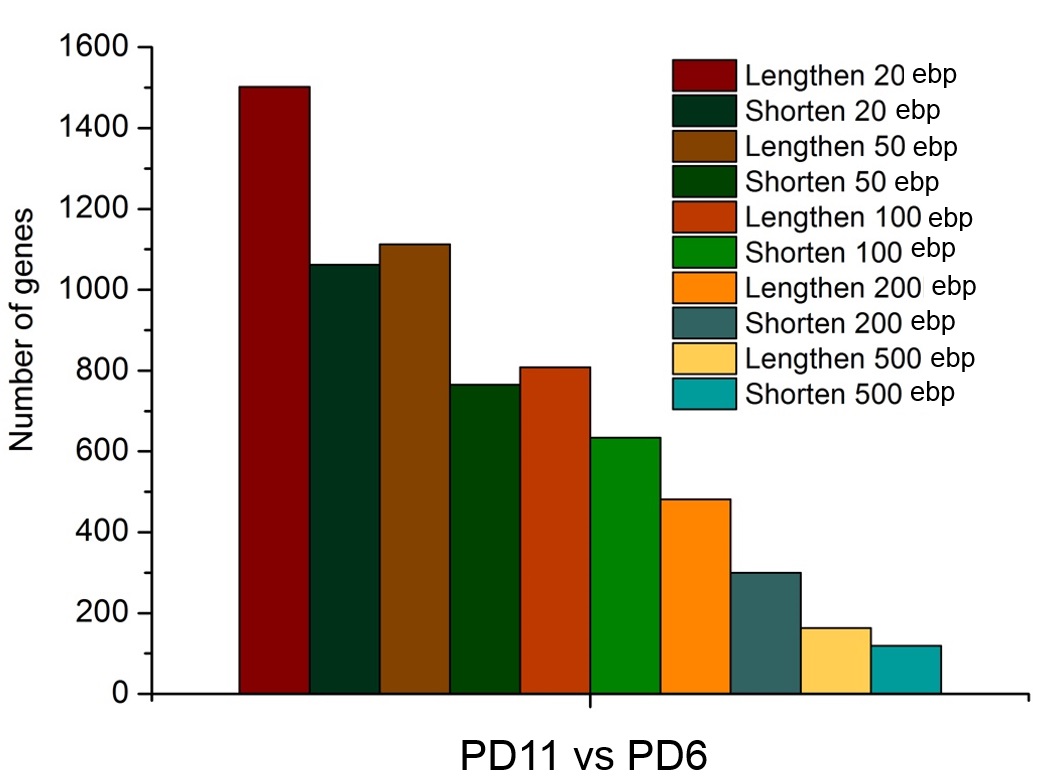


**Supplemental Figure S6. Biological replicate of senescent MEFs confirmed global lengthening of 3′ UTRs for genes with APA regulation.** Number of genes with lengthened effective 3′ UTRs and number of genes with shortened effective 3′ UTRs by comparing PD11 to PD6 given different thresholds based on PA-seq data.
