# Supplementary material for: 3′ UTR lengthening as a novel mechanism in regulating cellular senescence
Source: Genome Res. 2018 Mar;28(3):285–94. doi: 10.1101/gr.224451.117 (PMC5848608; doi:10.1101/gr.224451.117)
Supplement: Supplemental Material [file supp_gr.224451.117_Supplemental_Fig_S7.docx]

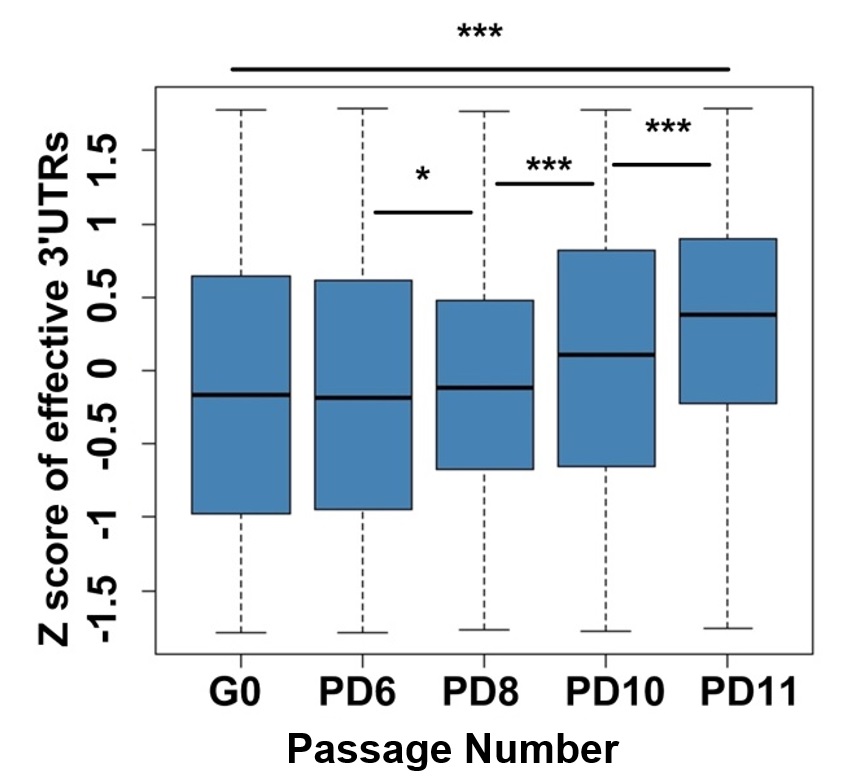


**Supplemental Figure 7. Box plot for Z-score transformed effective 3′ UTRs across G0, PD6, PD8, PD10, and PD11 of MEFs.** (***) P < 0.001 and (*) P < 0.05, two-tailed Wilcoxon signed rank test.


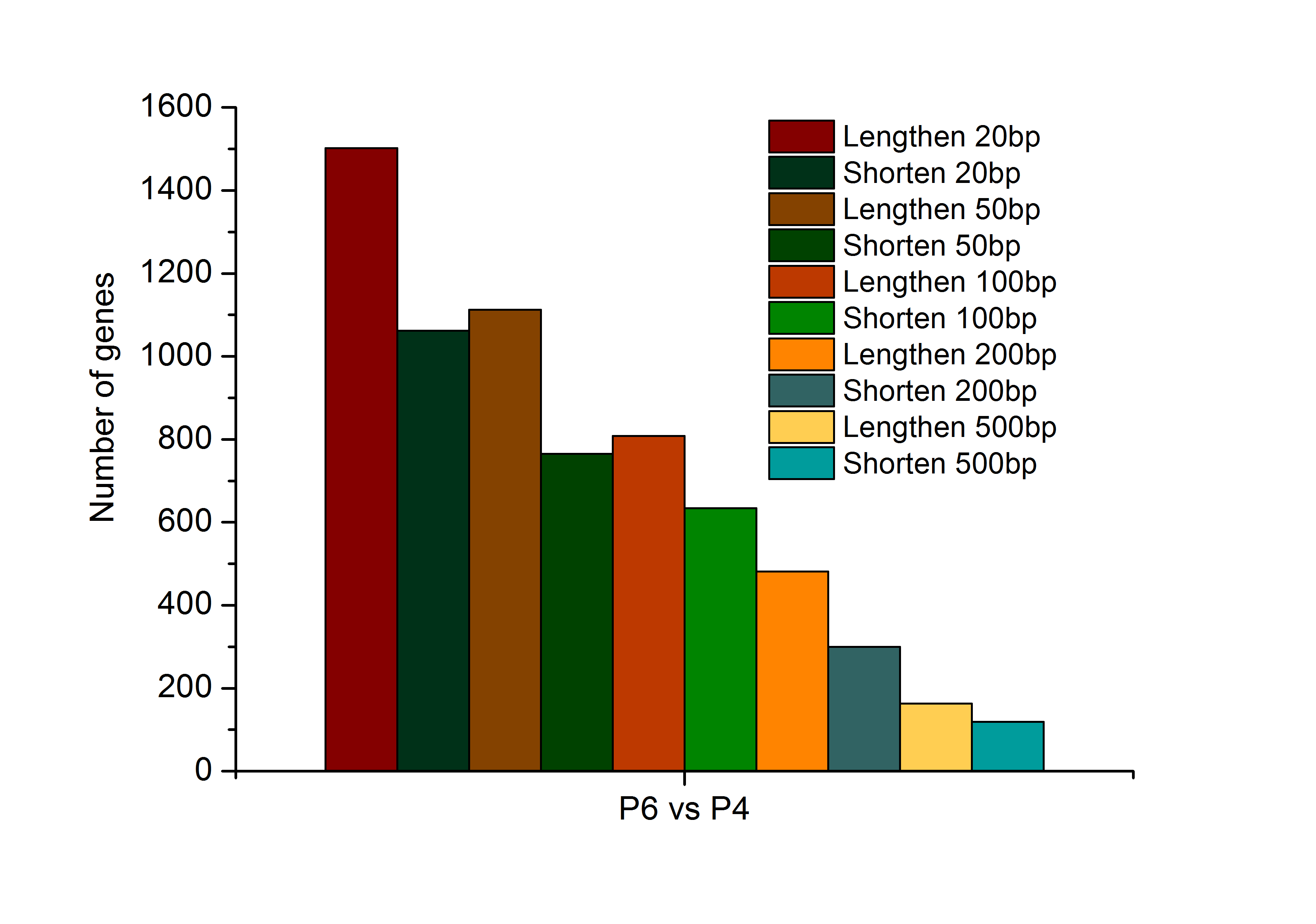
 PD11 vs PD6

**Supplemental Figure 8. Biological replicate of senescent MEFs confirmed global lengthening of 3′ UTRs for genes with APA regulation.** Number of genes with lengthened effective 3′ UTRs and number of genes with shortened effective 3′ UTRs by comparing PD11 to PD6 given different thresholds based on PA-seq data.

**
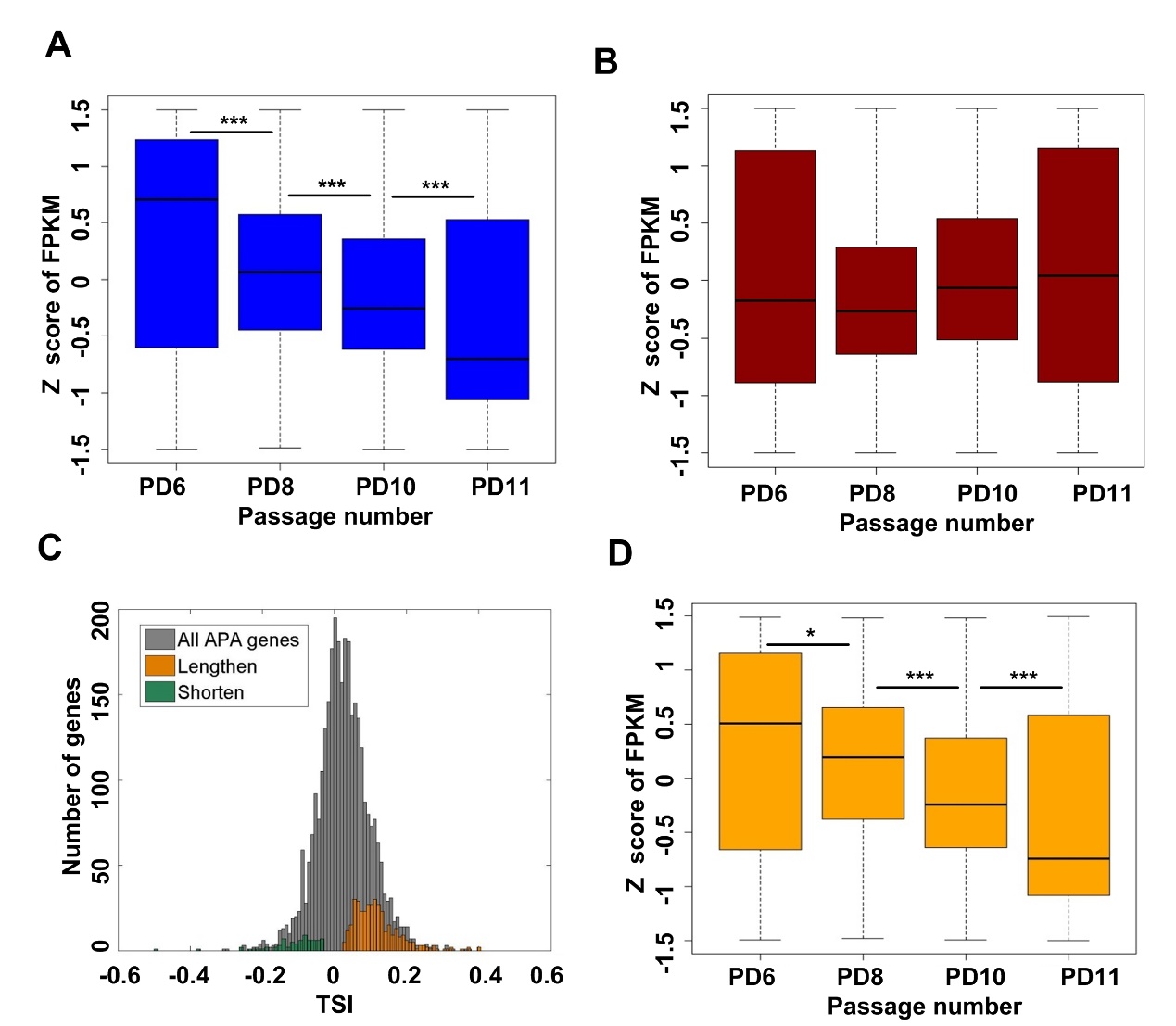
**

**Supplemental Figure 9. Genes preferred to use distal pAs in senescent cells tended to have decreased mRNA abundance.** (A) Box plot of Z-score transformed FPKM for genes with multiple pAs across PD6, PD8, PD10, and PD11. (B) Box plot of Z-score transformed FPKM for genes with single pA site across PD6, PD8, PD10, and PD11. (C) Distribution of TSI values for all APA involved genes. Orange and green bars refer to that for genes using continuous lengthened and shortened 3′ UTRs during replicative senescence in MEFs, respectively. TSI denotes tandem UTR isoform switch index values (see Online Methods for details). (D) Box plot of FPKM values across PD6, PD8, PD10, and PD11 for genes with progressively lengthened 3′ UTRs during replicative senescence in MEFs. (***) P < 0.001 and (*) P < 0.05, two-tailed Wilcoxon signed rank test.

**
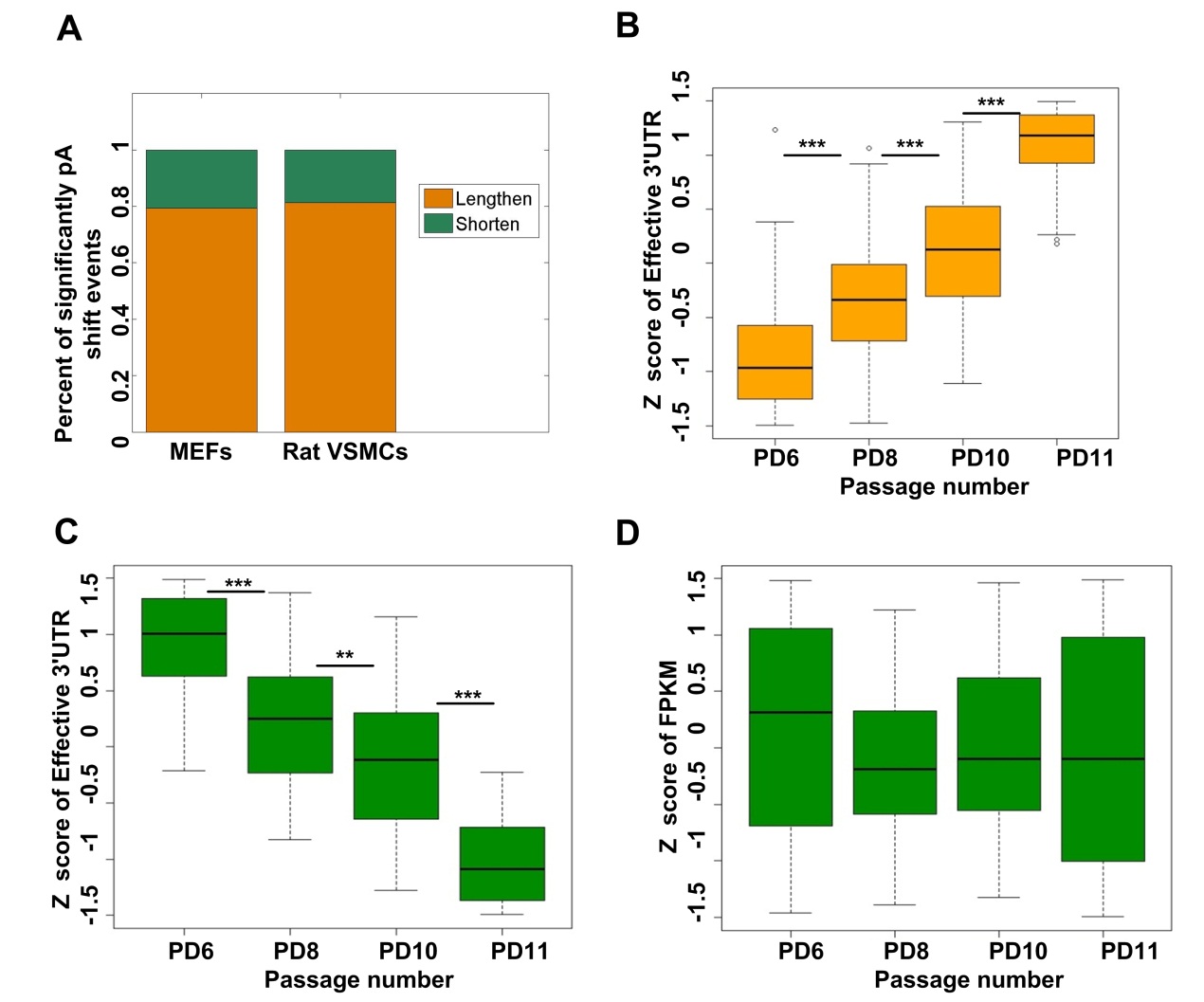
 Supplemental Figure 10. Genes tended to use proximal pAs did not have decreased mRNA abundance.** (A) Fraction of genes significantly tended to use distal pAs (lengthen) and proximal pAs (shorten) in senescent MEFs (PD11) comparing to early passage of MEFs (PD6), and also that in VSMCs from old rats (2 years old) comparing to young rats (2 weeks old). (B) Distribution of Z scores of effective 3′ UTRs across PD6, PD8, PD10, and PD11 for genes with lengthened 3′ UTRs during replicative senescence of MEFs. (C) Comparing distribution of Z scores of effective 3′ UTRs length across PD6, PD8, PD10, and PD11 for genes with shortened 3′ UTRs during replicative senescence of MEFs. (D). Distribution of Z-score transformed FPKM across PD6, PD8, PD10, and PD11 for genes with shortened 3′ UTRs during replicative senescence of MEFs. (***) *P*$<$0.001 and (**) *P*$<$0.01, two-tailed Wilcoxon signed rank test.


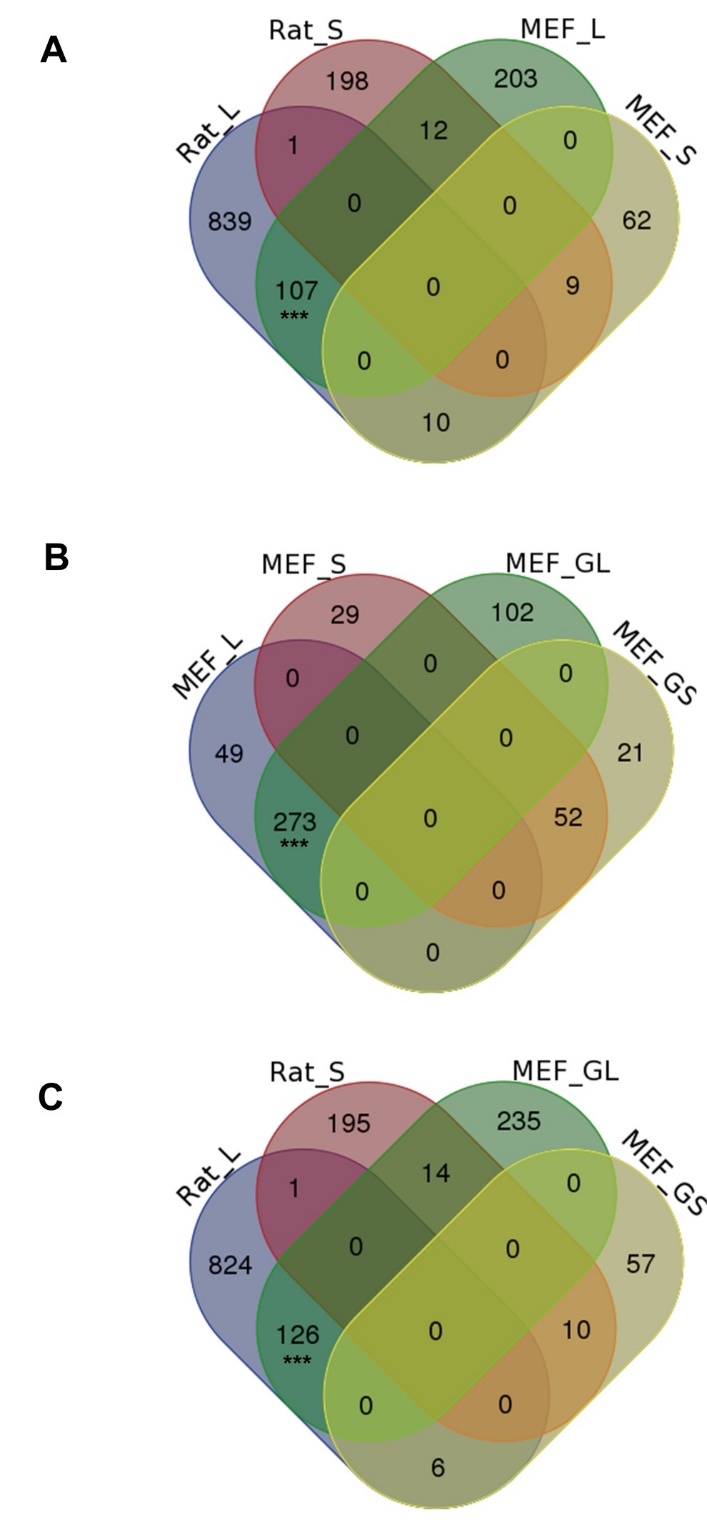


**Supplemental Figure 11. Comparison among genes significantly tended to use distal pAs and proximal pAs in senescent MEFs and aged rat VSMCs.** (A) Venn diagram for genes preferring distal (named Rat_L) and proximal (named Rat_S) pAs when comparing VSMCs of old rat with young rat, and genes preferring distal (MEF_L) and proximal (MEF_S) pAs when comparing senescent MEFs (PD11) with young MEFs (PD6). (B) Venn diagram comparison among MEF_L, MEF_S, and genes gradually preferred to use distal (MEF_GL) and proximal (MEF_GS) pAs during replicative senescence of MEFs. (C) Venn diagram comparison among Rat_L, Rat_S, MEF_GL, and MEF_GS. MEF_L, MEF_S, Rat_L, Rat_S, MEF_GL, and MEF_GS were identified by linear trend test with the Benjamini-Hochberg (BH) false-discovery rate (FDR) at 5%. (***) *P*$<$0.001, Fishers′ exact test.


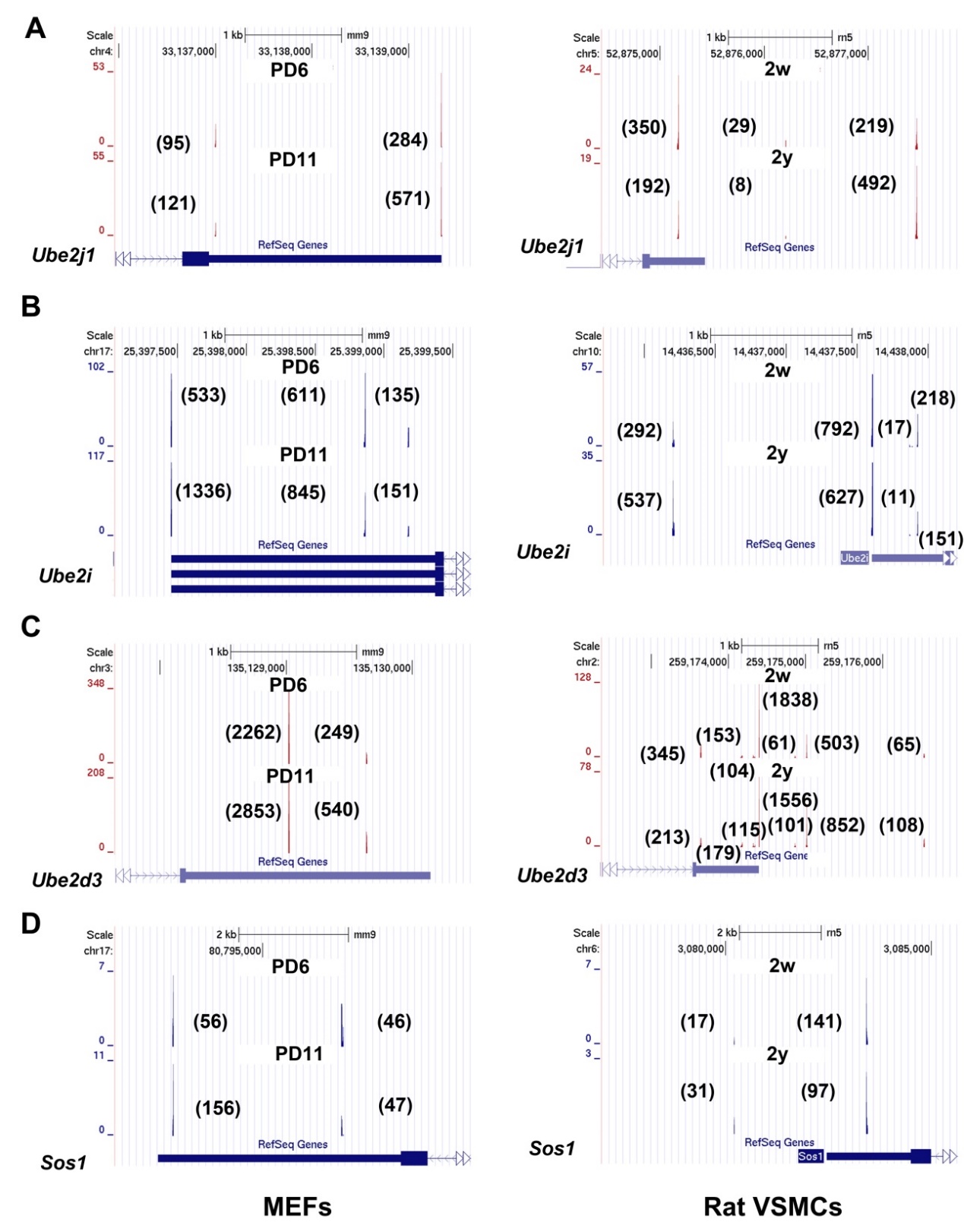


**Supplemental Figure 12. Examples of genes tended to use distal pAs in senescent MEFs and VSMCs of aged rat.** (A-D) PA-seq tracks of *Ube2j1*, *Ube2i*, *Ube2d3*, and *Sos1* in senescent and young MEFs and VSMCs from old and young rats. Numbers in brackets indicate the raw tag numbers for each pA site.


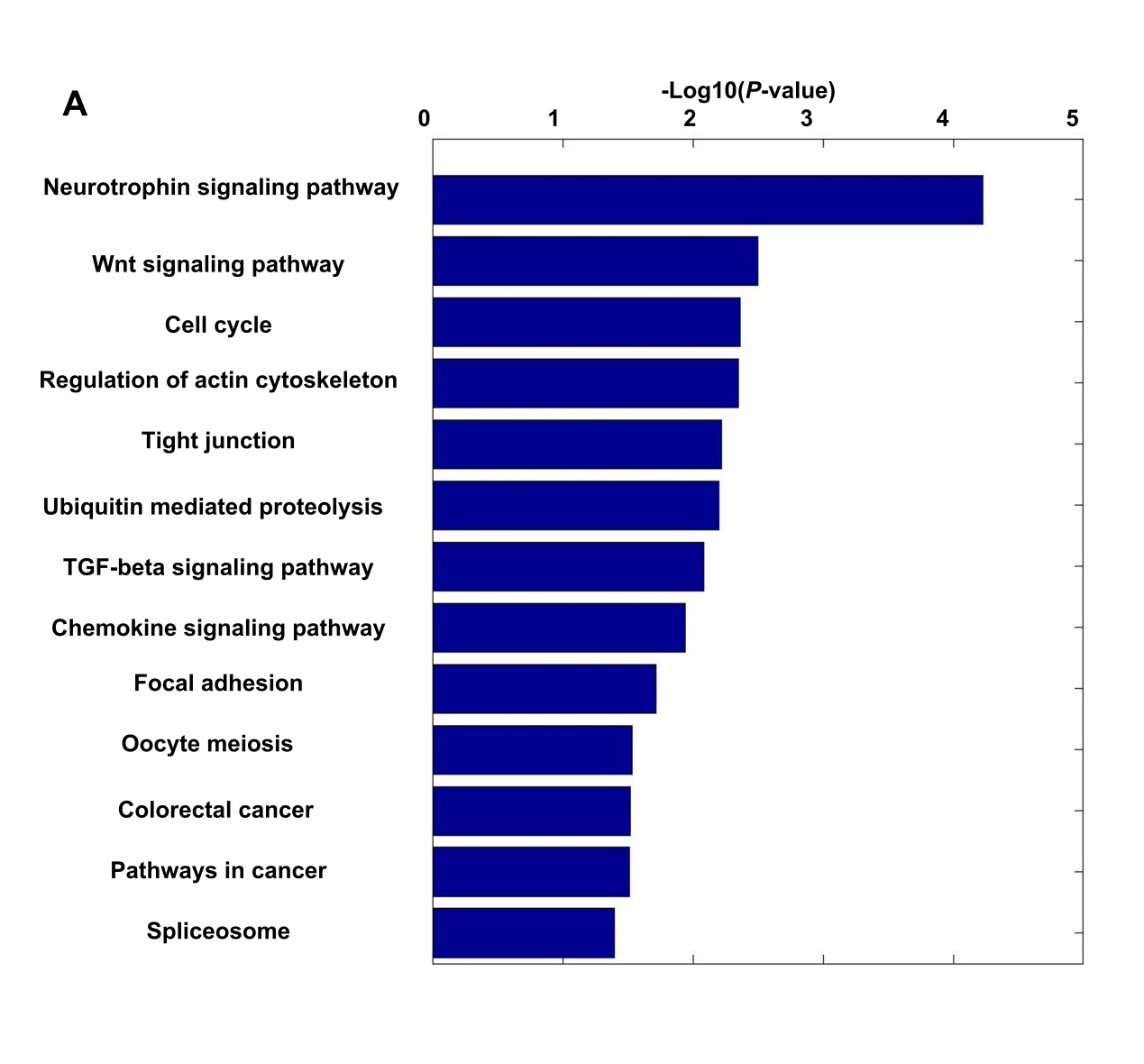


**Supplemental Figure 13. Genes progressively tend to use distal pAs during replicative senescence of MEFs are enriched in senescence-related pathways.**

**
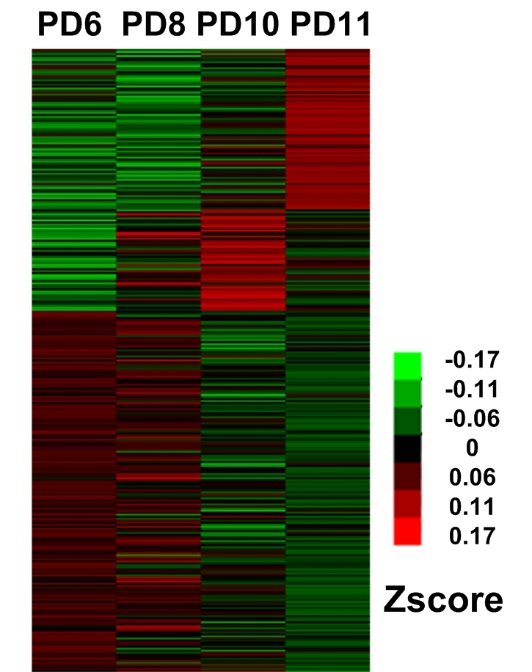
**

**Supplemental Figure 14. Heat map of RBPs expression during replicative senescence of MEFs.**

**
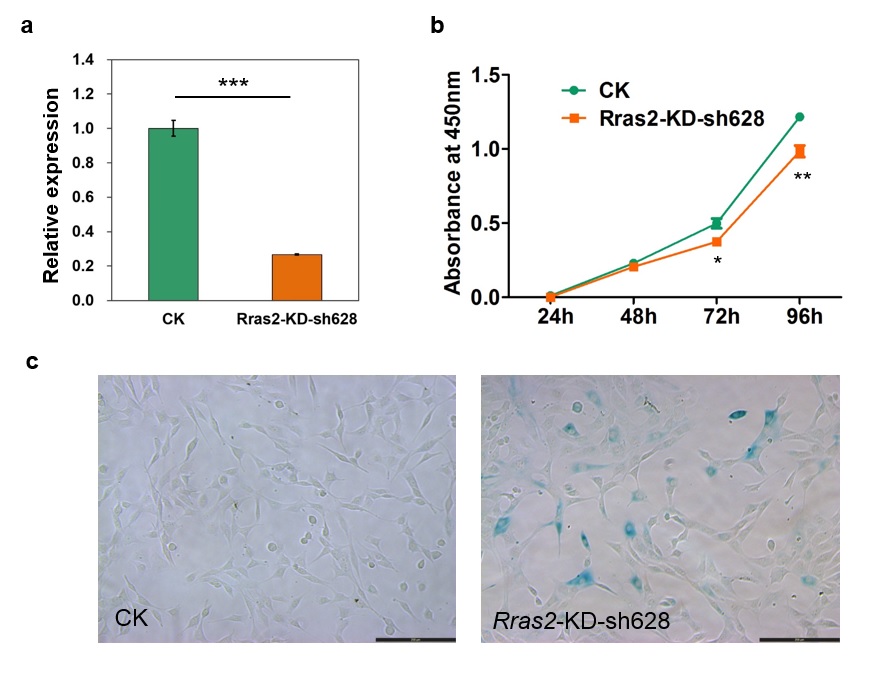
**

**Supplemental Figure 15. Knockdown of *Rras2* by shRNA sh628 leads to senescence in NIH3T3 cells.** (A) qRT-PCR results in control (CK) and KD NIH3T3 cells. (B-C) CCK-8 assay (B) and SA-β-gal staining (C) for control and KD NIH3T3 cells. Scale bar, 200 μm.

**
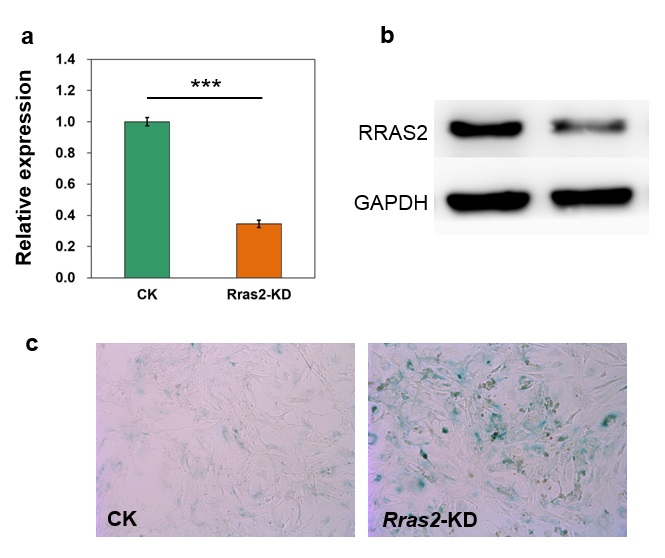
**

**Supplemental Figure 16. Knockdown of *Rras2* leads to higher SA-β-gal staining in primary MEF cells.** (A-B) qRT-PCR (A) and Western blot (B) in control (CK) and KD MEFs. (C) SA-β-gal staining for control and *Rras2*-KD MEFs. Scale bar, 200 μm.

**
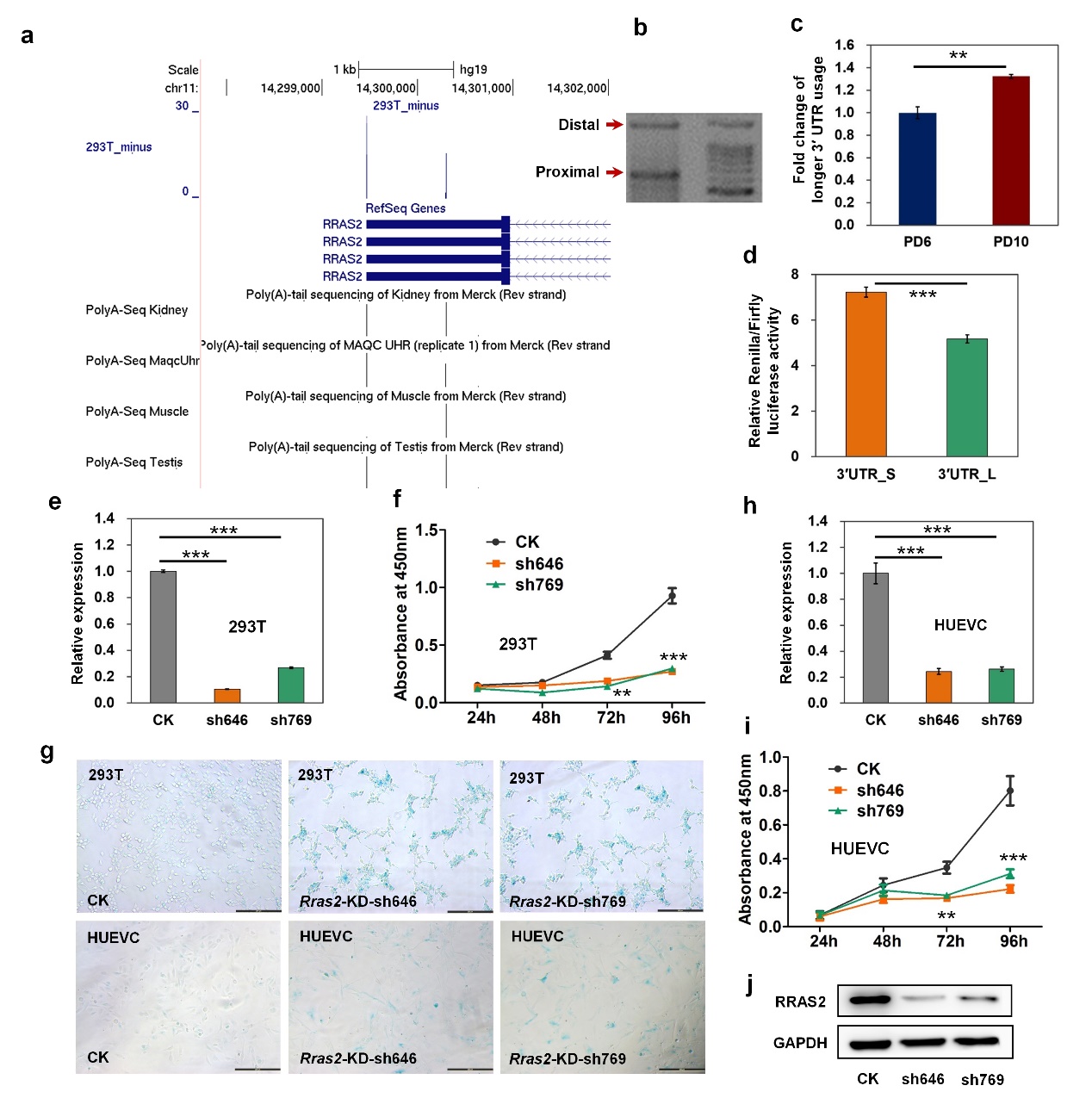
**

**Supplemental Figure 17. Decreased *Rras2* protein level via 3′ UTR lengthening causes senescence in human cells.** (A) UCSC genome browser shows PolyA-Seq track of *Rras2*. (B) 3′ RACE product in agarose gel to confirm distal and proximal pA sites in human cells. Right lane represents the molecular weight marker. (C) *Rras2* gene has higher usage of distal pA site in HUVEC PD10 than in PD6 determined by qRT-PCR. (D) Luciferase activity from a reporter containing the short 3′ UTR compared to that from the reporter containing the long 3′ UTR of *Rras2* (3′ UTR_S, and 3′ UTR_L, respectively). (E,H) Validation of *Rras2* knockdown by two different shRNAs by qRT-PCR in HEK293T (E) and HUVEC (H) cells. (F,I) Cell Counting Kit-8 (CCK-8) analysis to evaluate proliferation rate of HEK293T (F) and HUEVC (I) cells without (CK) and with *Rras2* knockdown (sh646 and sh769). (G) SA-β-gal staining for both HEK293T and HUEVC cells without (CK) and with *Rras2* knockdown (*Rras2*-KD). Scale bar, 200 μm. (J) Western blot confirms RRAS2 knockdown efficiency in HUEVC cells. GAPDH serves as loading control. (***) P < 0.001, (**) P < 0.01, two-tailed t-test.

**
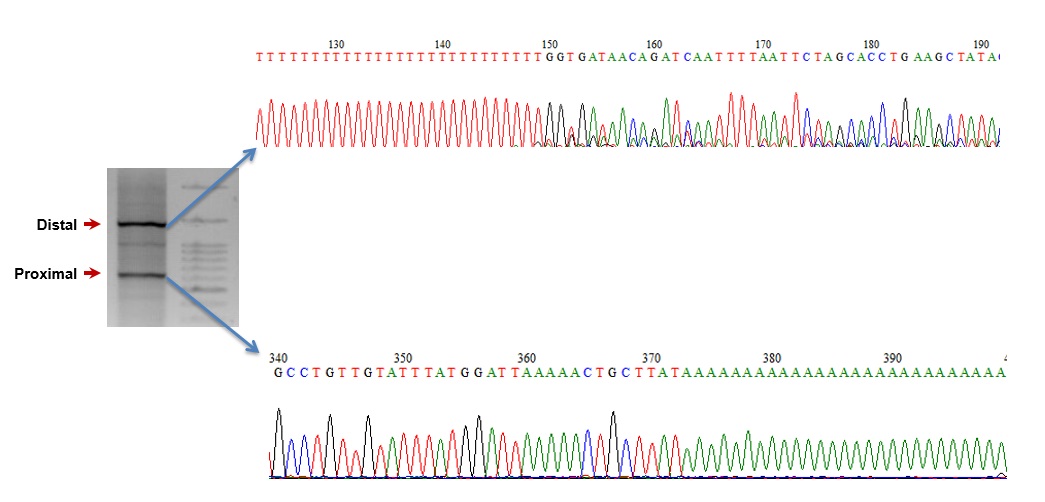
**

**Supplemental Figure 18. Sanger sequencing of 3′ RACE products derived from proximal (bottom) or distal (top) pA sites in human HUVEC cells.**


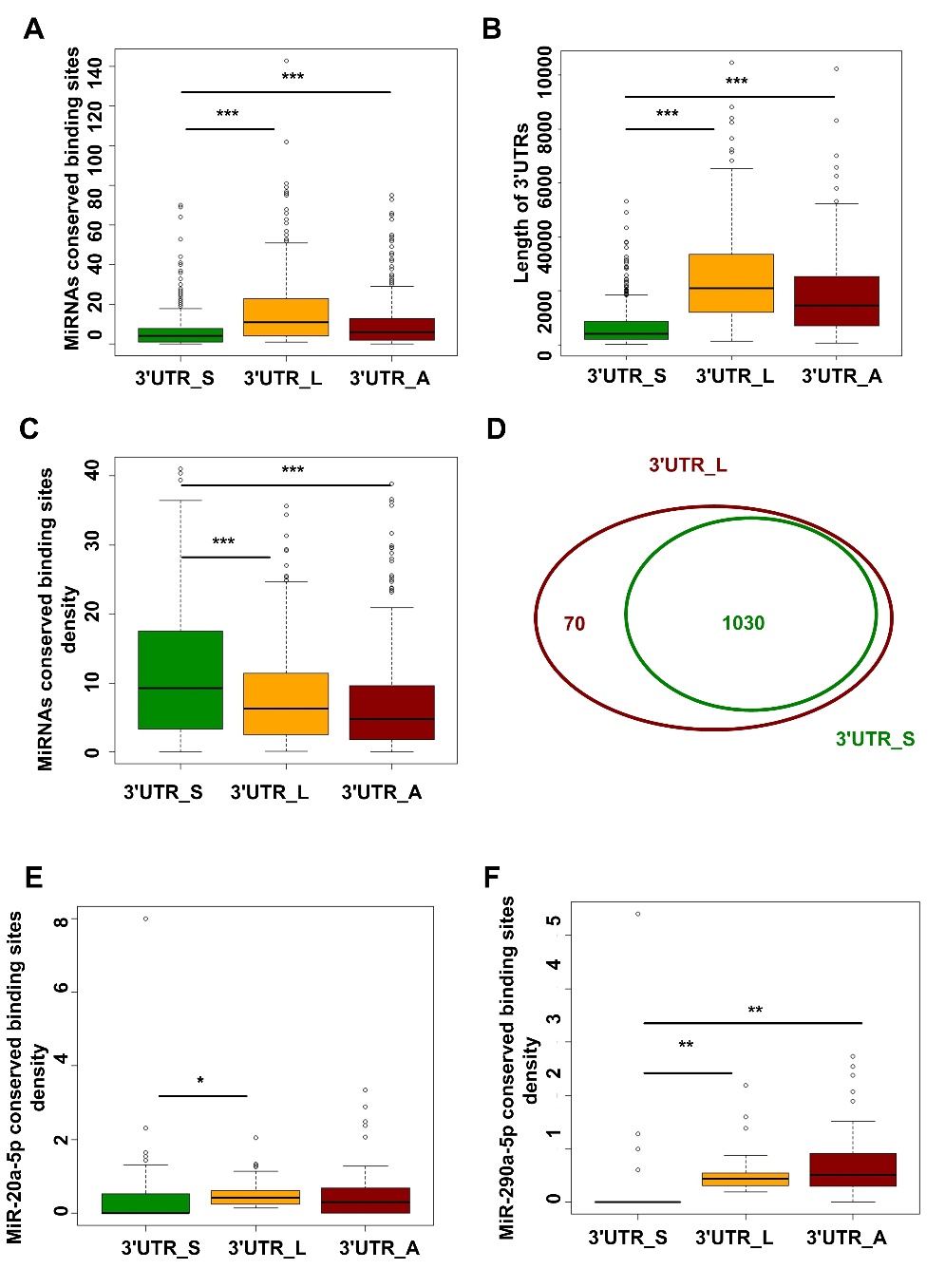


**Supplemental Figure 19. Genes progressively tend to use distal pAs during replicative senescence of MEFs can introduce more conserved miRNA binding sites.** (A) Box plot comparison for the number of conserved miRNA binding sites among the shortest 3′ UTRs (3′UTR_S), the longest 3′ UTRs (3′UTR_L), and alternative 3′ UTRs (3′UTR_A) in genes that tend to progressively use distal pAs during replicative senescence of MEFs. (B) Box plot for length comparison among 3′ UTR_S, 3′UTR_L and 3′UTR_A. (C) Box plot comparison for density of conserved miRNA binding sites among 3′ UTR_S, 3′UTR_L, and 3′UTR_A. (D) Venn diagram comparing conserved miRNA binding sites in the 3′ UTR_S with 3′ UTR_L. (E) Comparing the density of miR-20a-5p potential binding sites in 3′ UTR_S with 3′ UTR_L and 3′ UTR_A. (F) Comparing the density of miR-290a-5p potential binding sites in 3′ UTR_S with 3′ UTR_L and 3′ UTR_A. (***) *P*$<$0.001, (**) *P*$<$0.01 and (*) *P*$<$0.05, two-tailed Wilcoxon signed rank test.


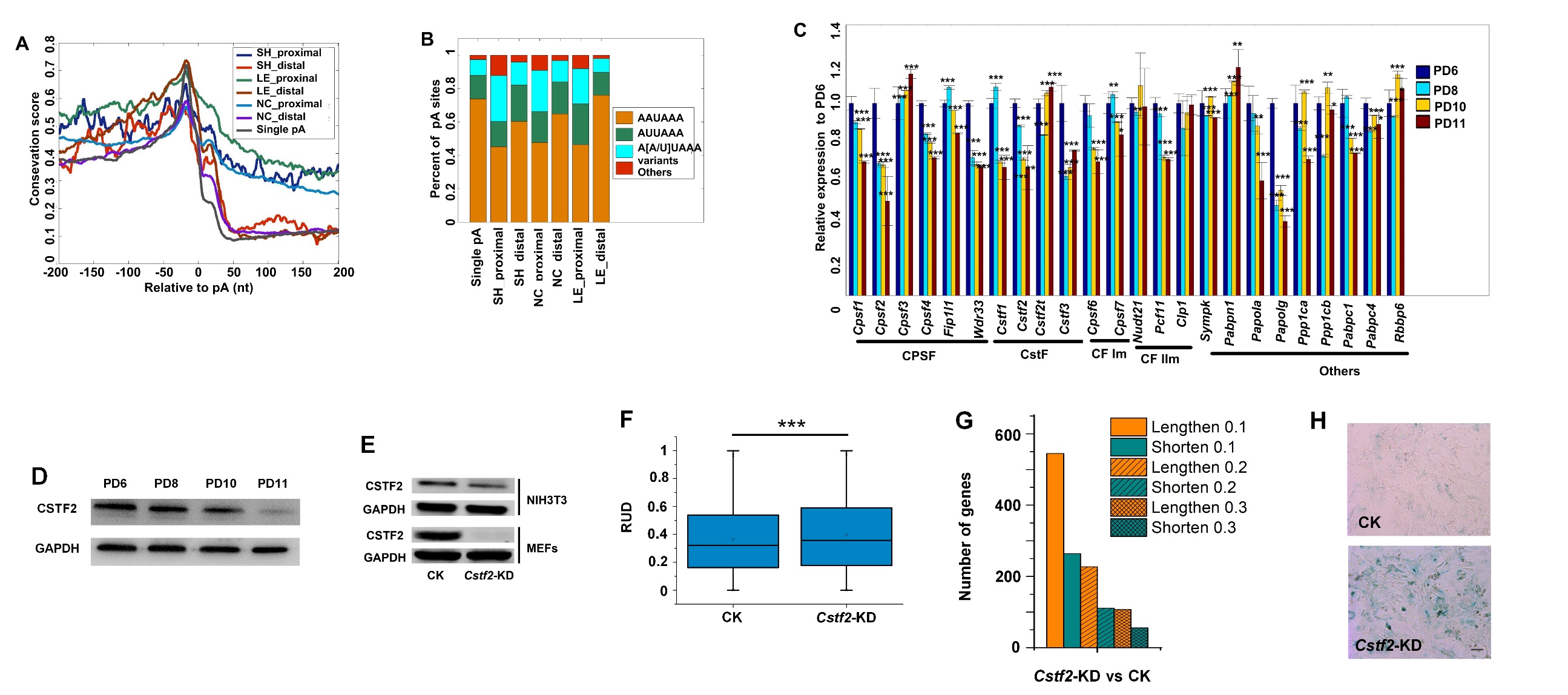


**Supplemental Figure 20. DNA sequence analyses for APA regulation during replicative senescence of MEFs.** (A) Comparison of conservation score in the 400 nt region surrounding different types of pAs. (B) Distribution of PAS sequences in the -40 to -1 nt region for different categories of pAs.


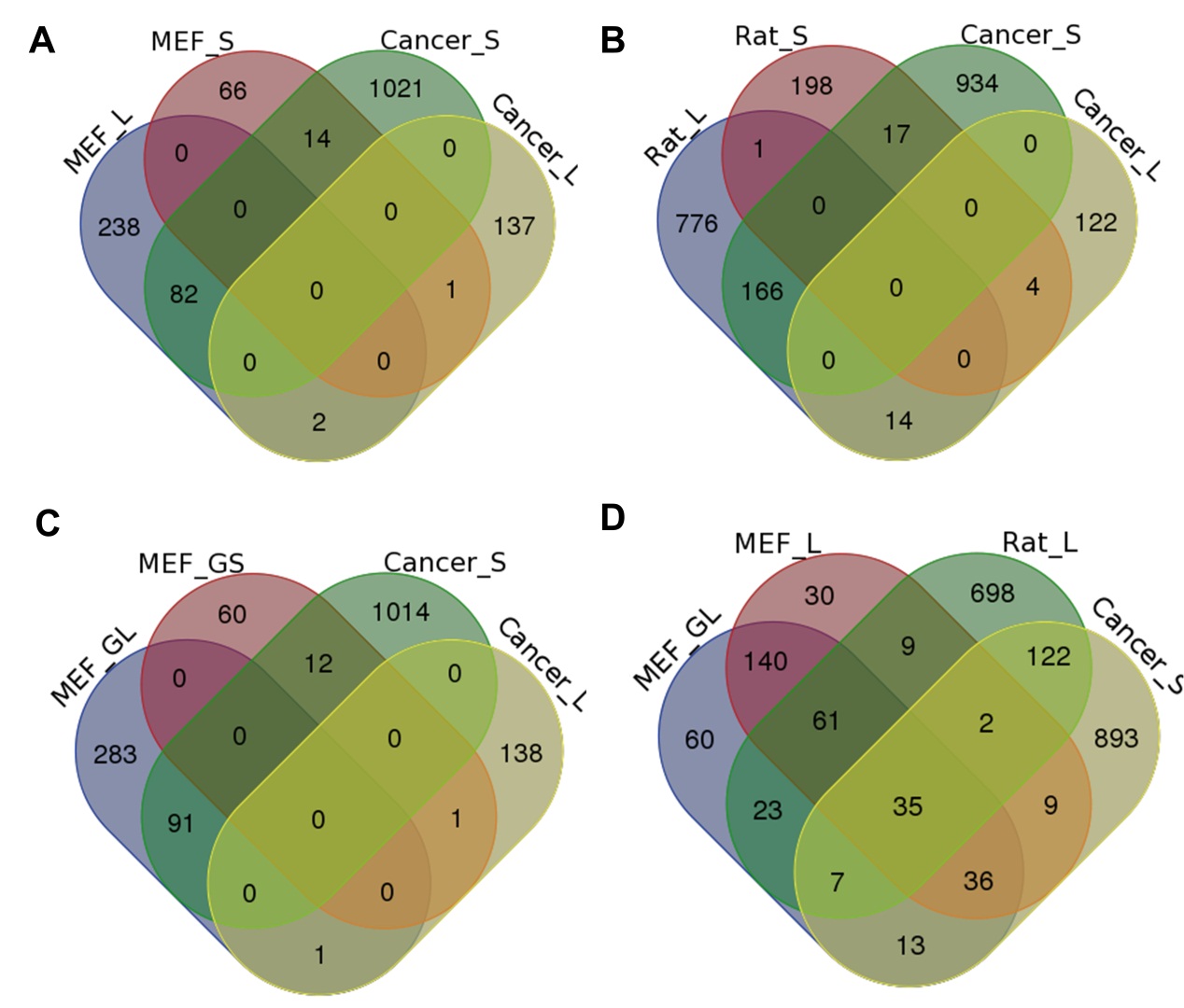


**Supplemental Figure 21. Comparison of genes that tended to use distal and proximal pAs between senescent and cancer cells.** (A) Venn diagram comparison among genes preferring distal (MEF_L) and proximal (MEF_S) pAs when comparing senescent MEFs (PD11) with young MEFs (PD6), and genes favoring distal (Cancer_L) and proximal (Cancer_S) pAs when comparing tumors and normal tissues identified by Xia et al. [3]. (B) Venn diagram comparison among genes preferring distal (Rat_L) and proximal (Rat_S) pAs when comparing VSMCs of old rat with young rat, Cancer_L, and Cancer_S. (C) Venn diagram comparison among genes gradually preferred to use distal (MEF_GL) and proximal (MEF_GS) pAs during replicative senescence of MEFs, Cancer_L, and Cancer_S. (D). Venn diagram comparison among MEF_L, Rat_L, MEF_GL, and Cancer_S. MEF_L, MEF_S, Rat_L, Rat_S, MEF_GL, and MEF_GS were identified by a linear trend test with the Benjamini-Hochberg (BH) false-discovery rate (FDR) at 5%.

**Supplemental Table 1. MEFs PA-seq** **reads mapping statistics.**

| **Sample** | **Total Reads** | **Mapped Read1** | **Read1 mapping rate** | **Mapped Read2** | **Read2 mapping rate** |
| --- | --- | --- | --- | --- | --- |
| G0 | 15,411,083 | 9,442,546 | 61.3% | 11,471,857 | 74.4% |
| PD6 | 9,423,643 | 4,478,586 | 47.5% | 5,689,182 | 60.4% |
| PD8 | 13,386,797 | 7,822,028 | 58.4% | 9,472,369 | 70.8% |
| PD10 | 8,779,338 | 3,818,641 | 43.5% | 4,799,825 | 54.7% |
| PD11 | 14,425,358 | 8,848,238 | 63.3% | 10,736,847 | 74.4% |

**Supplemental Table 2: 18,639 Refined pAs identified in this study and their assigned categories (available as a separate Excel file).**

**Supplemental Table 3. MEFs RNA-seq reads mapping statistics.**

| **Sample** | **Total Reads** | **Mapped Read1** | **Read1 mapping rate** | **Mapped Read2** | **Read2 mapping rate** |
| --- | --- | --- | --- | --- | --- |
| G0 | 15,809,150 | 13,997,188 | 88.5% | 15,809,150 | 88.6% |
| PD6 | 28,132,487 | 27,048,742 | 96.1% | 26,876,587 | 95.5% |
| PD8 | 25,659,880 | 24,321,086 | 94.8% | 24,175,499 | 94.2% |
| PD10 | 17,860,083 | 16,254,722 | 91.0% | 16,060,448 | 89.9% |
| PD11 | 16,396,313 | 14,101,035 | 86.0% | 13,980,835 | 85.3% |

**Supplemental Table 4: Summary 3,165 genes with APA regulation (available as a separate Excel file).**

**Supplemental Table 5. qRT-PCR primers used to validate genes with significant switch of APA usage.**

| **Gene** | **Common region primers  (from 5′ to 3′)** | **Alternative region primers  (from 5′ to 3′)** |
| --- | --- | --- |
| *Daam2* | GAGCGGGCCAATAAACAGG  CCTTTCTTCTAATCCCTGTCTCC | TTCAGGGGTTGGAAGGACAA  TTGCTTGTCTTCTGCAGCAG |
| *Anapc1* | CCTAGAAATGACTGCGGCAC  CATATGACACGTGGACAGCA | TGCCATGGGAGGACTTGAAA  GCTGTGCATGGTAAGACTGG |
| *Map3k7* | ACAGCAGGCTAATCAGGAGG  GGCAACAGACTCAGGAAAGG | GTTGGCACTCACTCACTTGG  GACCAGAGCTCACTTCCTGT |
| *Ccnh* | AGGAATGGACTGATGACGACC  CAGACATGCTTCCTACTTCCG | GCAAACCATGTCCTCCTGTG  TTGTAAGGGCTTCTGGAGGA |
| *Fbxo28* | TTCAGTTTTCTTTGGGGCCG  TGACAACTCTCTCCCGACAG | TGCCGATCCACACAGTCATA  AGACTCCACCATTCTCCAGC |
| *Gadd45b* | AGGTGGCCAGTTACTGTGAA  TTTAGGGGACAGCAACTCGA | GGAGACTGAGACTTTAGAGCCA  CTCCGCTGACTTATGCACAG |
| *Ube2b* | TCCAAACAGTCCAGCCAACA  TCCTTAAAACCCGTGGCACT | CCAGCTCTGTATTTGGGCCA  TCTCTGCCTTGCTCAAACCA |
| *Tceb2* | CATCGAGCCCTTTTCCAGC  GGGAAATGGGTCTCTAGGGG | AGGATTCTGGAGGCAGTGC  ACTTGGGGTTAAGAGTTCTGTG |
| *Ctsb* | AGGACAAATGCCACCTCTCA  GAGGACAGGACCAAGGAGAG | AGTATGAGTGCCAGGCCTTT  GGCTCTGTGGTAGTGGAAGT |
| *Acvr2a* | TGGAAAGCATGGATCTGGGA  TCCTTGATTTGGAGAGGGCC | CCTTGCCCAAATCTCCCATG  GTGCAAGTTCATGGGACCAA |
| *Sec61g* | TGCTTAAACGTGACTGCTTTTC  TTTACTTCATGCCCTTTCCCC | GGGTTGGCTGAGTCCTTCT  AGAAACAAAACACCACACAGC |
| *Lamc1* | TCCATCGAGAAGCCCTAGTG  AGTGATGGAGAGCAGCAGAG | ACTGTGGCCCTTTTCAATGT  AAAATGCCAAGTGTTGCGCT |
| *Cul1* | AACAGACGCCAATGCCATTT  TCCATTCAGACTCGCTCTCG | TAACAGCTGTCGTCTGAGGC  ACCAAATGCAACTTGTACAGAAA |

**Supplemental Table 6. PA-seq reads mapping statistics for biological replicate of MEF.**

| **Sample** | **Total Reads** | **Mapped Read1** | **Read1 mapping rate** | **Mapped Read2** | **Read2 mapping rate** |
| --- | --- | --- | --- | --- | --- |
| PD6 | 17,309,769 | 12,532,955 | 72.4% | 13,647,511 | 78.8% |
| PD11 | 26,553,217 | 17,115,583 | 64.5% | 18,725,415 | 70.5% |

**Supplemental Table 7. Rat PA-seq reads mapping statistics.**

| **Sample** | **Total Reads** | **Strand specific reads** | **Mapped Read1** | **Read1 mapping rate** | **Mapped Read2** | **Read2 mapping rate** |
| --- | --- | --- | --- | --- | --- | --- |
| 2 weeks | 26,122,246 | 20,468,514 | 8,818,888 | 43.1% | 11,694,741 | 57.1% |
| 2 years | 35,087,625 | 29,152,131 | 12,443,319 | 42.7% | 16,098,918 | 55.2% |

**Supplemental Table 8. List of genes with significant pA usage shift in replicative senescence of MEFs and aortic vascular smooth muscle cells of rats (VSMCs) at different ages. (Available as a separate Excel file)**

**Supplemental Table 9. Functional enrichment analysis for genes with significantly pA usage shift in replicative senescence of MEFs and aortic vascular smooth muscle cells of rats (VSMCs) at different ages. (Available as a separate Excel file).**

**Supplemental Table 10. Primer sequences for qRT-PCR, luciferase assay and shRNA (Available as a separate Excel file).**

**References in Supplemental Materials**
