# Supplementary material for: 3′ UTR lengthening as a novel mechanism in regulating cellular senescence
Source: Genome Res. 2018 Mar;28(3):285–94. doi: 10.1101/gr.224451.117 (PMC5848608; doi:10.1101/gr.224451.117)
Supplement: Supplemental Material [file supp_gr.224451.117_Supplemental_Fig_S8.docx]

**
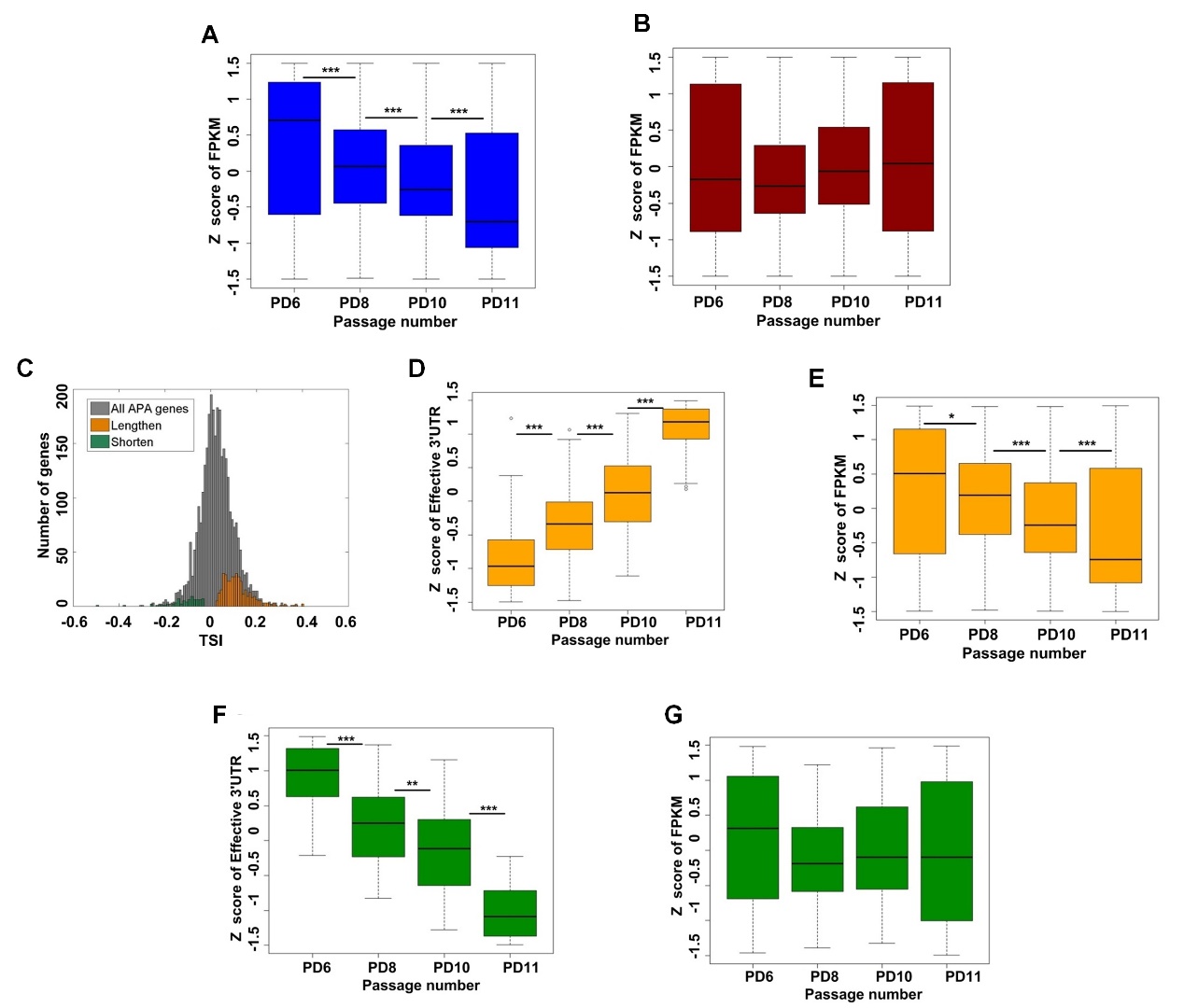
**

**Supplemental Figure S8. Genes preferred to use distal pAs in senescent cells tended to have decreased mRNA abundance.** (A) Box plot of Z-score transformed FPKM for genes with multiple pAs across PD6, PD8, PD10, and PD11. (B) Box plot of Z-score transformed FPKM for genes with single pA site across PD6, PD8, PD10, and PD11. No expression decrease trend was observed during senescence. (C) Distribution of TSI values for all APA involved genes. Orange and green bars refer to that for genes using continuous lengthened and shortened 3′ UTRs during replicative senescence in MEFs, respectively. TSI denotes tandem UTR isoform switch index values (see Online Methods for details). (D) Distribution of Z scores of effective 3′ UTRs across PD6, PD8, PD10, and PD11 for genes with lengthened 3′ UTRs during replicative senescence of MEFs (orange labeled genes in panel C). (E) Box plot of FPKM values across PD6, PD8, PD10, and PD11 for genes with progressively lengthened 3′ UTRs (orange labeled genes in panel C) during replicative senescence in MEFs. (F) Comparing distribution of Z scores of effective 3′ UTRs length across PD6, PD8, PD10, and PD11 for genes with shortened 3′ UTRs during replicative senescence of MEFs. (G) Distribution of Z-score transformed FPKM across PD6, PD8, PD10, and PD11 for genes with shortened 3′ UTRs during replicative senescence of MEFs in panel F. (***) P < 0.001 and (*) P < 0.05, two-tailed Wilcoxon signed rank test.
