# Supplementary material for: 3′ UTR lengthening as a novel mechanism in regulating cellular senescence
Source: Genome Res. 2018 Mar;28(3):285–94. doi: 10.1101/gr.224451.117 (PMC5848608; doi:10.1101/gr.224451.117)
Supplement: Supplemental Material [file supp_gr.224451.117_Supplemental_Fig_S9.docx]

**
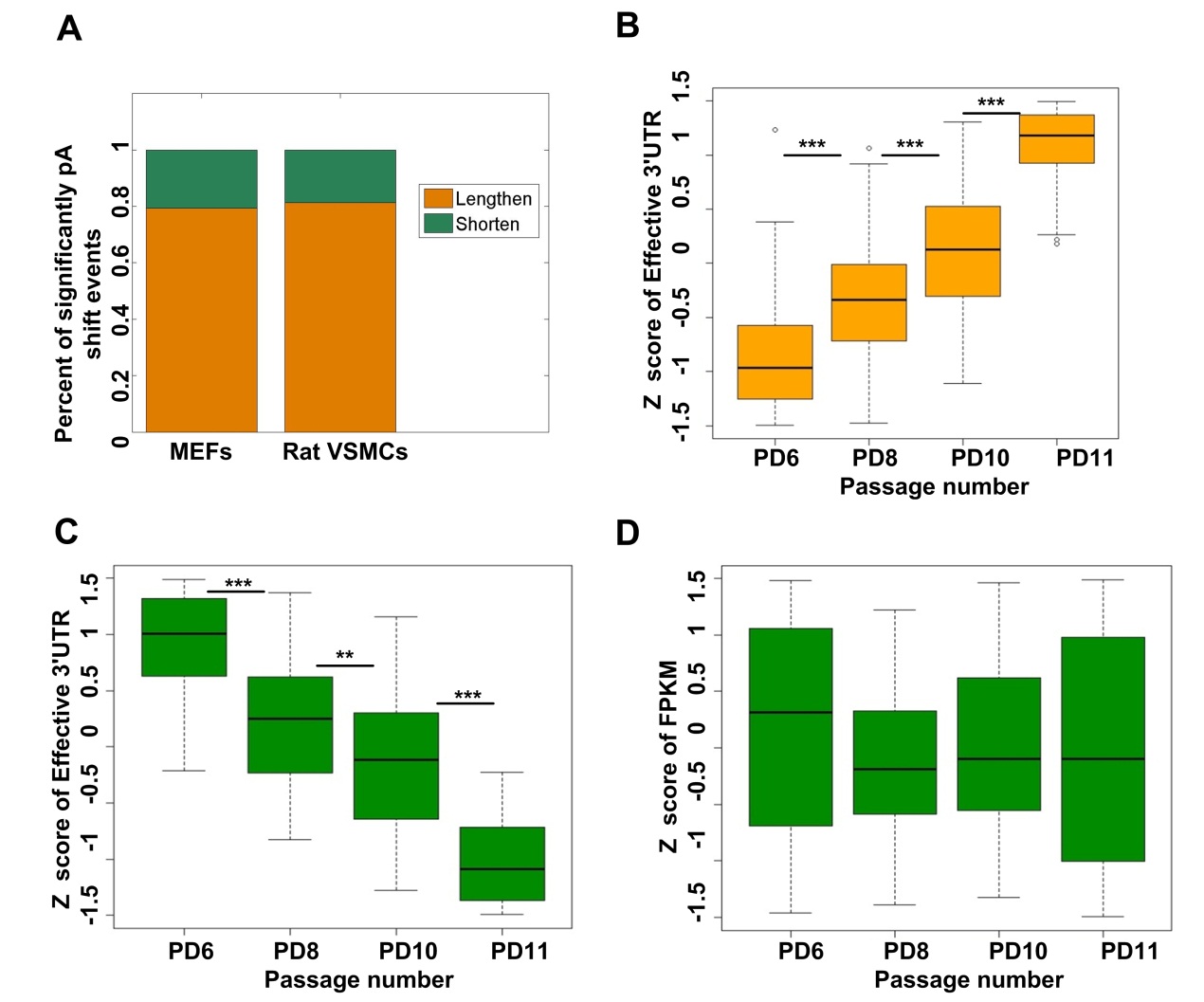
**

**Supplemental Figure S9.** Fraction of genes significantly tended to use distal pAs (lengthen) and proximal pAs (shorten) in senescent MEFs (PD11) comparing to early passage of MEFs (PD6), and also that in VSMCs from old rats (2 years old) comparing to young rats (2 weeks old).
