# Supplementary material for: 3′ UTR lengthening as a novel mechanism in regulating cellular senescence
Source: Genome Res. 2018 Mar;28(3):285–94. doi: 10.1101/gr.224451.117 (PMC5848608; doi:10.1101/gr.224451.117)
Supplement: Supplemental Material [file supp_gr.224451.117_Supplemental_Fig_S10.docx]

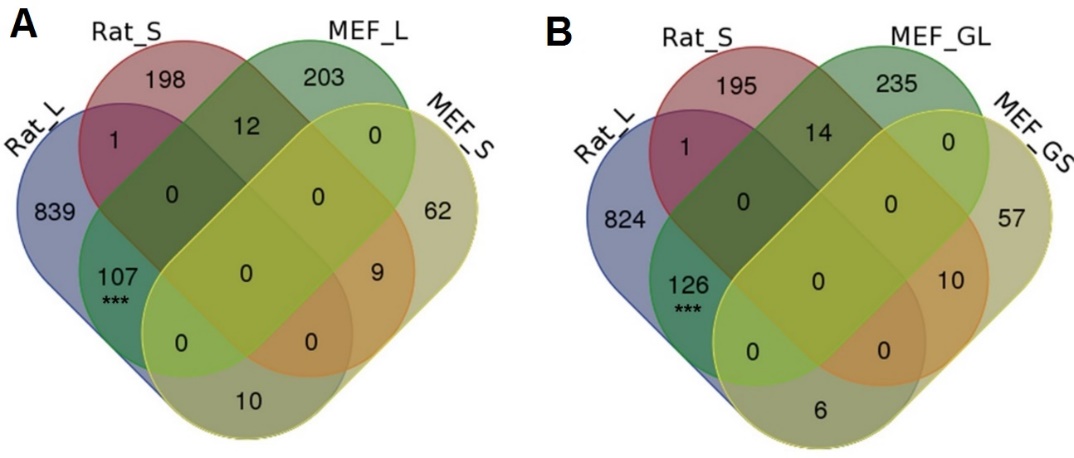


**Supplemental Figure S10. Comparison among genes significantly tended to use distal pAs and proximal pAs in senescent MEFs and aged rat VSMCs.** (A) Venn diagram for genes preferring distal (named Rat_L) and proximal (named Rat_S) pAs when comparing VSMCs of old rat with young rat, and genes preferring distal (MEF_L) and proximal (MEF_S) pAs when comparing senescent MEFs (PD11) with young MEFs (PD6). (B) Venn diagram comparison among Rat_L, Rat_S, and genes gradually preferred to use distal (MEF_GL) and proximal (MEF_GS) pAs during replicative senescence of MEFs. MEF_L, MEF_S, Rat_L, Rat_S, MEF_GL, and MEF_GS were identified by linear trend test with the Benjamini-Hochberg (BH) false-discovery rate (FDR) at 5%. (***) *P*$<$0.001, Fishers′ exact test.
