# Supplementary material for: 3′ UTR lengthening as a novel mechanism in regulating cellular senescence
Source: Genome Res. 2018 Mar;28(3):285–94. doi: 10.1101/gr.224451.117 (PMC5848608; doi:10.1101/gr.224451.117)
Supplement: Supplemental Material [file supp_gr.224451.117_Supplemental_Fig_S11.docx]

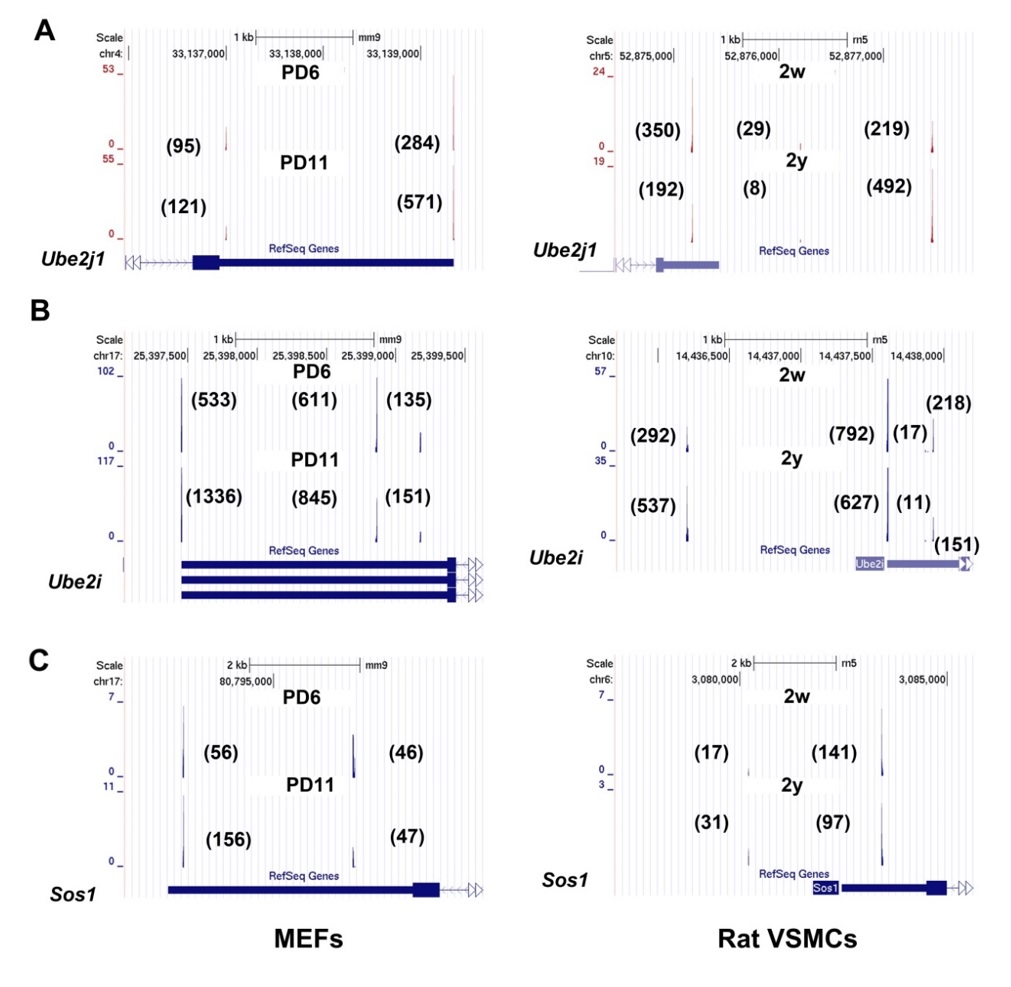


**Supplemental Figure S11. Examples of genes tended to use distal pAs in senescent MEFs and VSMCs of aged rat.** (A-C) PA-seq tracks of *Ube2j1*, *Ube2i*, and *Sos1* in senescent and young MEFs and VSMCs from old and young rats. Numbers in brackets indicate the raw tag numbers for each pA site.
