# Supplementary material for: 3′ UTR lengthening as a novel mechanism in regulating cellular senescence
Source: Genome Res. 2018 Mar;28(3):285–94. doi: 10.1101/gr.224451.117 (PMC5848608; doi:10.1101/gr.224451.117)
Supplement: Supplemental Material [file supp_gr.224451.117_Supplemental_Fig_S12.docx]

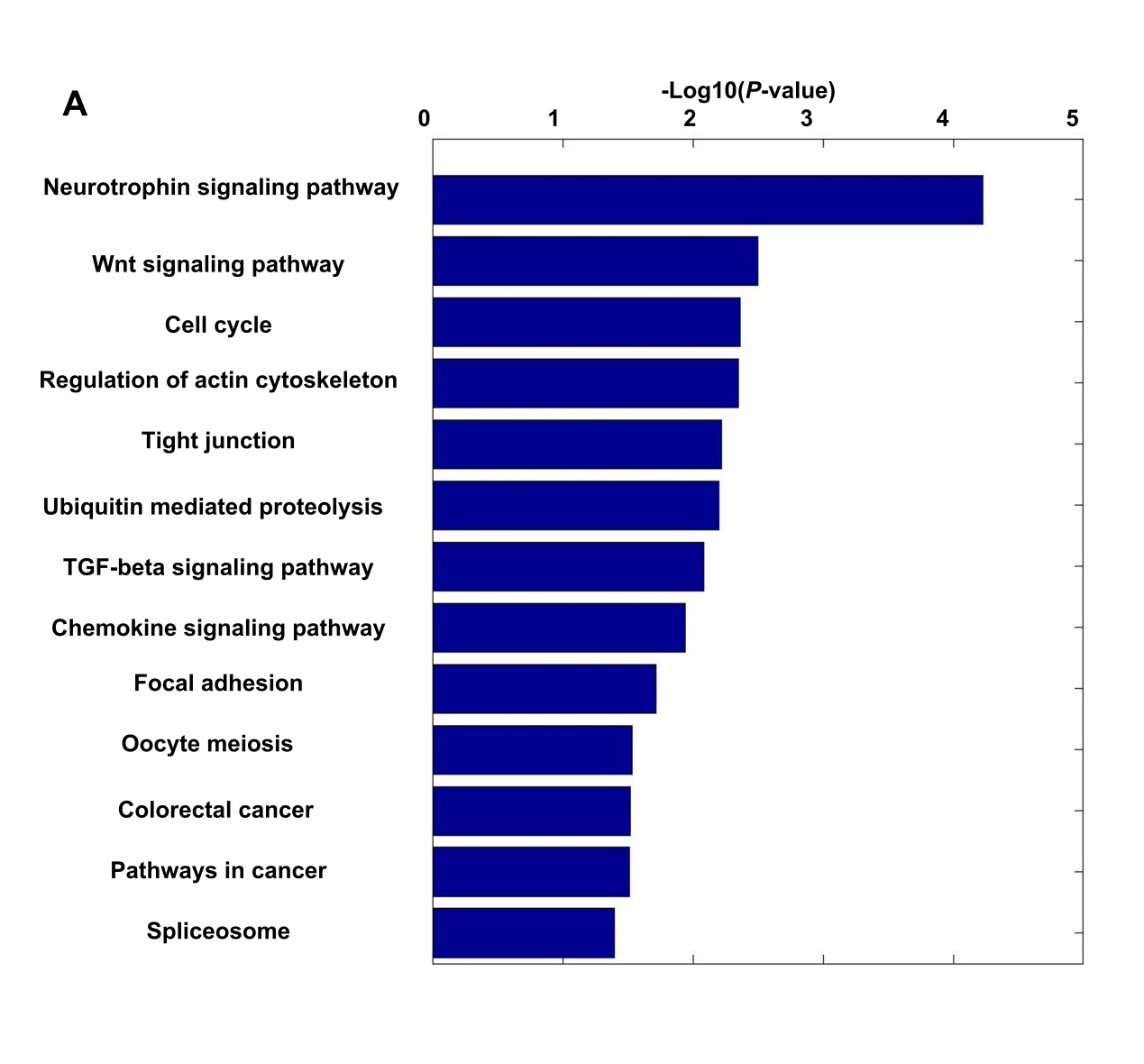


**Supplemental Figure S12. Genes progressively tend to use distal pAs during replicative senescence of MEFs are enriched in senescence-related pathways.** Genes with progressively lengthened 3′ UTRs were shown in Supplemental Fig. S8C.
