# Supplementary material for: 3′ UTR lengthening as a novel mechanism in regulating cellular senescence
Source: Genome Res. 2018 Mar;28(3):285–94. doi: 10.1101/gr.224451.117 (PMC5848608; doi:10.1101/gr.224451.117)
Supplement: Supplemental Material [file supp_gr.224451.117_Supplemental_Fig_S13.docx]

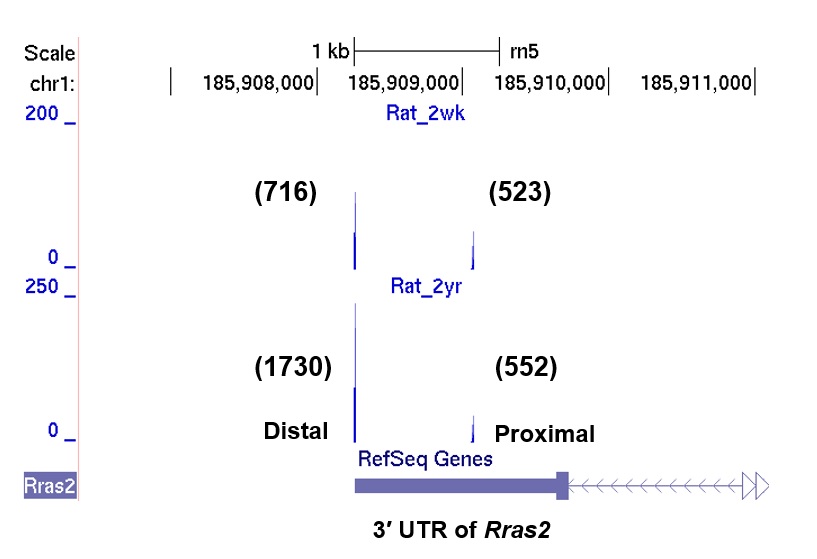


**Supplemental Figure S13. PA-seq track of proximal and distal pA site usage in VMSCs from 2-year (2yr) and 2-week (2wk) old rat.** Raw tag number for each pA site was indicated in parentheses.
