# Supplementary material for: 3′ UTR lengthening as a novel mechanism in regulating cellular senescence
Source: Genome Res. 2018 Mar;28(3):285–94. doi: 10.1101/gr.224451.117 (PMC5848608; doi:10.1101/gr.224451.117)
Supplement: Supplemental Material [file supp_gr.224451.117_Supplemental_Fig_S14.docx]

**
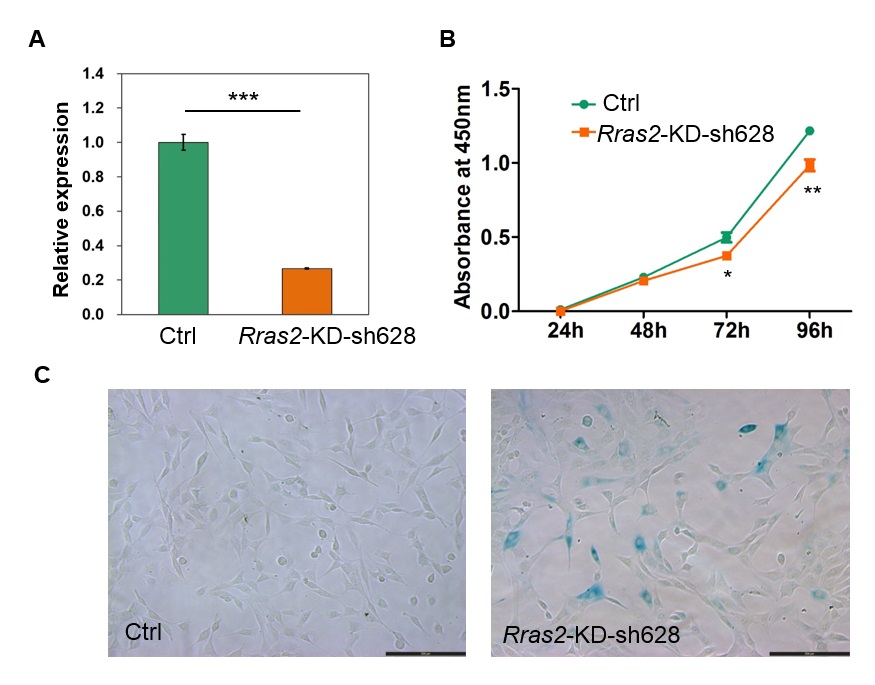
**

**Supplemental Figure S14. Knockdown of *Rras2* by shRNA sh628 leads to senescence in NIH3T3 cells.** (A) qRT-PCR results in control (Ctrl) and KD NIH3T3 cells. (B-C) CCK-8 assay (B) and SA-β-gal staining (C) for control and KD NIH3T3 cells. Scale bar, 200 μm.
