# Supplementary material for: 3′ UTR lengthening as a novel mechanism in regulating cellular senescence
Source: Genome Res. 2018 Mar;28(3):285–94. doi: 10.1101/gr.224451.117 (PMC5848608; doi:10.1101/gr.224451.117)
Supplement: Supplemental Material [file supp_gr.224451.117_Supplemental_Fig_S15.docx]

**
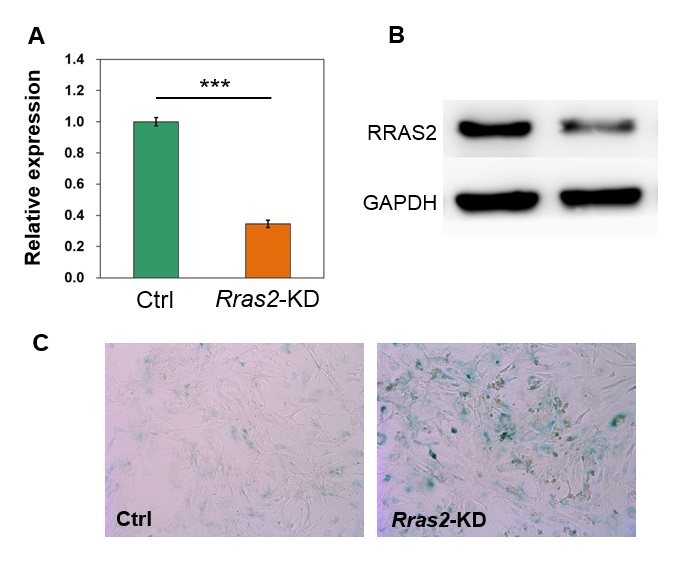
**

**Supplemental Figure S15. Knockdown of *Rras2* leads to higher SA-β-gal staining in primary MEF cells.** (A-B) qRT-PCR (A) and Western blot (B) in control (Ctrl) and KD (sh979) MEFs. (C) SA-β-gal staining for control and *Rras2*-KD MEFs. Scale bar, 200 μm.
