# Supplementary material for: 3′ UTR lengthening as a novel mechanism in regulating cellular senescence
Source: Genome Res. 2018 Mar;28(3):285–94. doi: 10.1101/gr.224451.117 (PMC5848608; doi:10.1101/gr.224451.117)
Supplement: Supplemental Material [file supp_gr.224451.117_Supplemental_Fig_S16.docx]

**
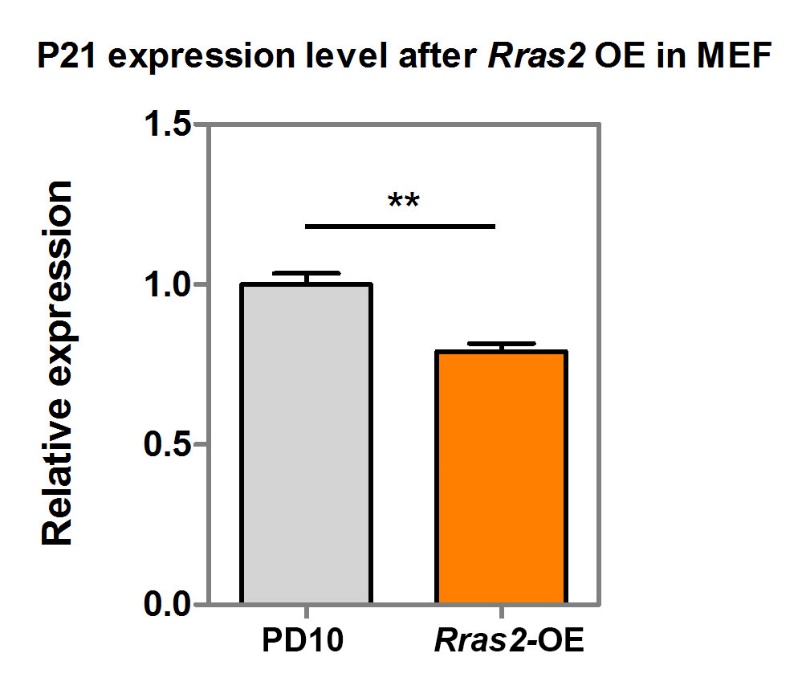
**

**Supplemental Figure S16. Overexpression of *Rras2* led to decreased *Cdkn1a* in primary MEF cells by qRT-PCR.** ** represents p value < 0.01, *t*-test.
