# Supplementary material for: 3′ UTR lengthening as a novel mechanism in regulating cellular senescence
Source: Genome Res. 2018 Mar;28(3):285–94. doi: 10.1101/gr.224451.117 (PMC5848608; doi:10.1101/gr.224451.117)
Supplement: Supplemental Material [file supp_gr.224451.117_Supplemental_Fig_S17.docx]

**
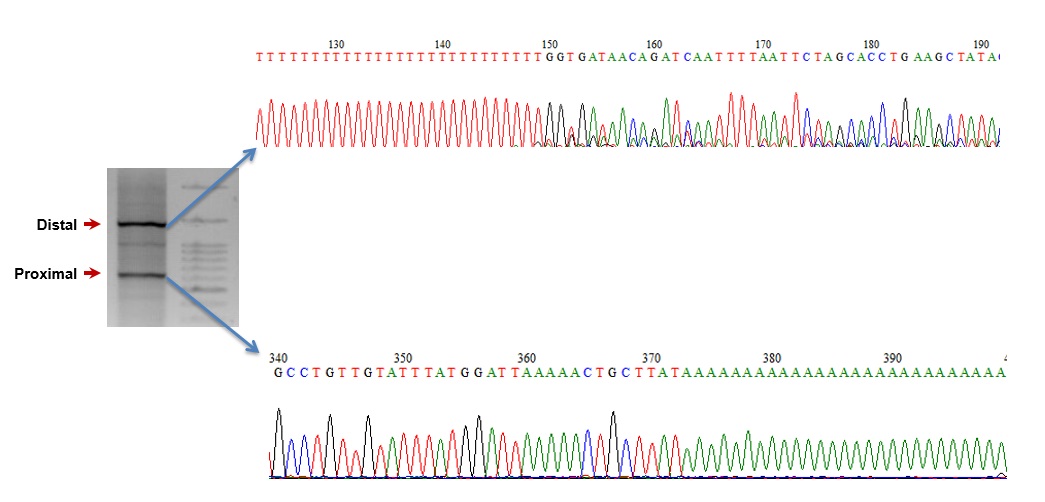
**

**Supplemental Figure S17. Sanger sequencing of 3′ RACE products derived from proximal (bottom) or distal (top) pA sites in human HUVEC cells.**
