# Supplementary material for: 3′ UTR lengthening as a novel mechanism in regulating cellular senescence
Source: Genome Res. 2018 Mar;28(3):285–94. doi: 10.1101/gr.224451.117 (PMC5848608; doi:10.1101/gr.224451.117)
Supplement: Supplemental Material [file supp_gr.224451.117_Supplemental_Fig_S18.docx]

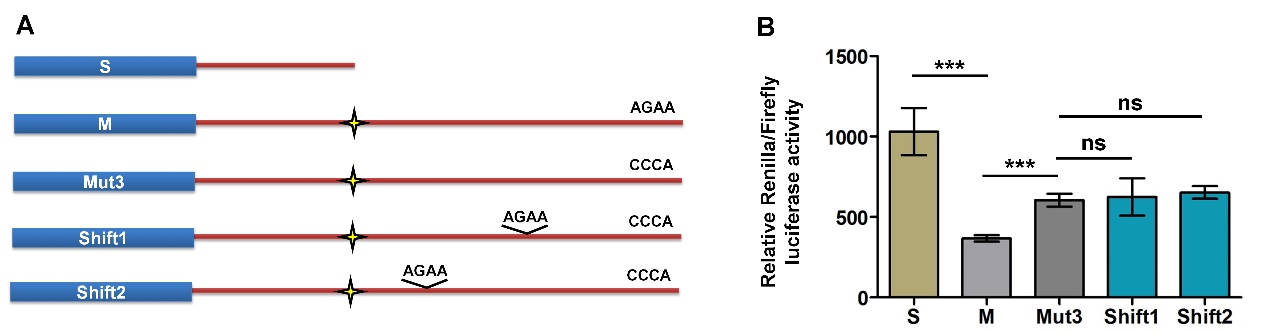


**Supplemental Fig. 18. Dual luciferase activity assay for the ‘AGAA’ motif shift in the alternative 3′-UTR of *Rras2*.** (A) ‘AGAA’ was mutated to ‘CCCA’ in the Mut3 construct. The ‘AGAA’ motif was introduced into two different locations in the alternative 3′-UTR of *Rras2*, which was labeled as ‘shift1’ and ‘shift2’. M denotes the full length 3′-UTR of *Rras2* with a mutated proximal polyA signal to force usage of only the distal pA site. (B) Relative *Renilla*/Firefly luciferase activity of constructs indicated in panel A. ** and *** represent p values of less than 0.01 and 0.001, respectively, *t*-test. ns denotes not significant.
