# Supplementary material for: 3′ UTR lengthening as a novel mechanism in regulating cellular senescence
Source: Genome Res. 2018 Mar;28(3):285–94. doi: 10.1101/gr.224451.117 (PMC5848608; doi:10.1101/gr.224451.117)
Supplement: Supplemental Material [file supp_gr.224451.117_Supplemental_Fig_S19.docx]

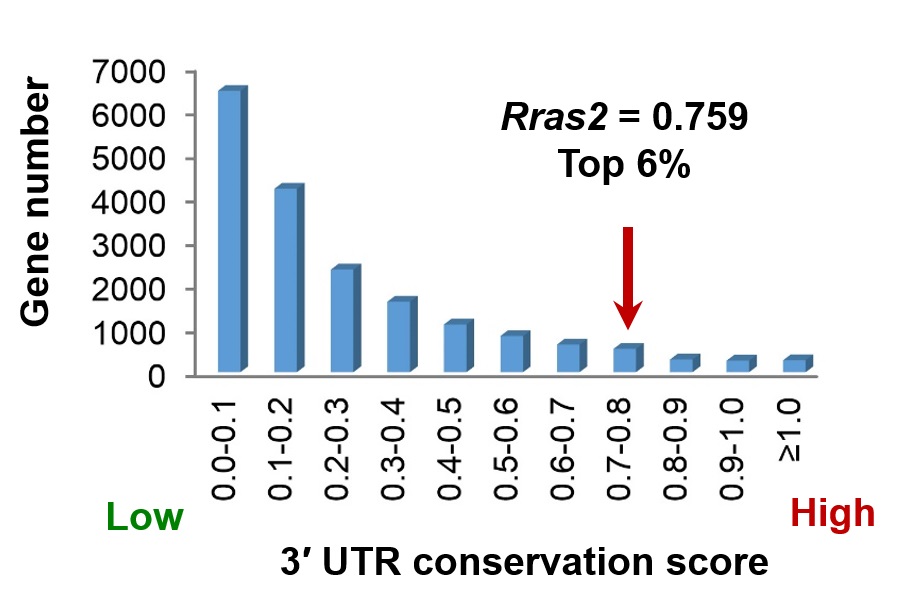


**Supplemental Figure S19. 3′ UTR of *Rras2* ranks top 6% regarding sequence conservation in 100 species based on Vertebrate Multiz Alignment & Conservation (UCSC).**
