# Supplementary material for: 3′ UTR lengthening as a novel mechanism in regulating cellular senescence
Source: Genome Res. 2018 Mar;28(3):285–94. doi: 10.1101/gr.224451.117 (PMC5848608; doi:10.1101/gr.224451.117)
Supplement: Supplemental Material [file supp_gr.224451.117_Supplemental_Fig_S20.docx]

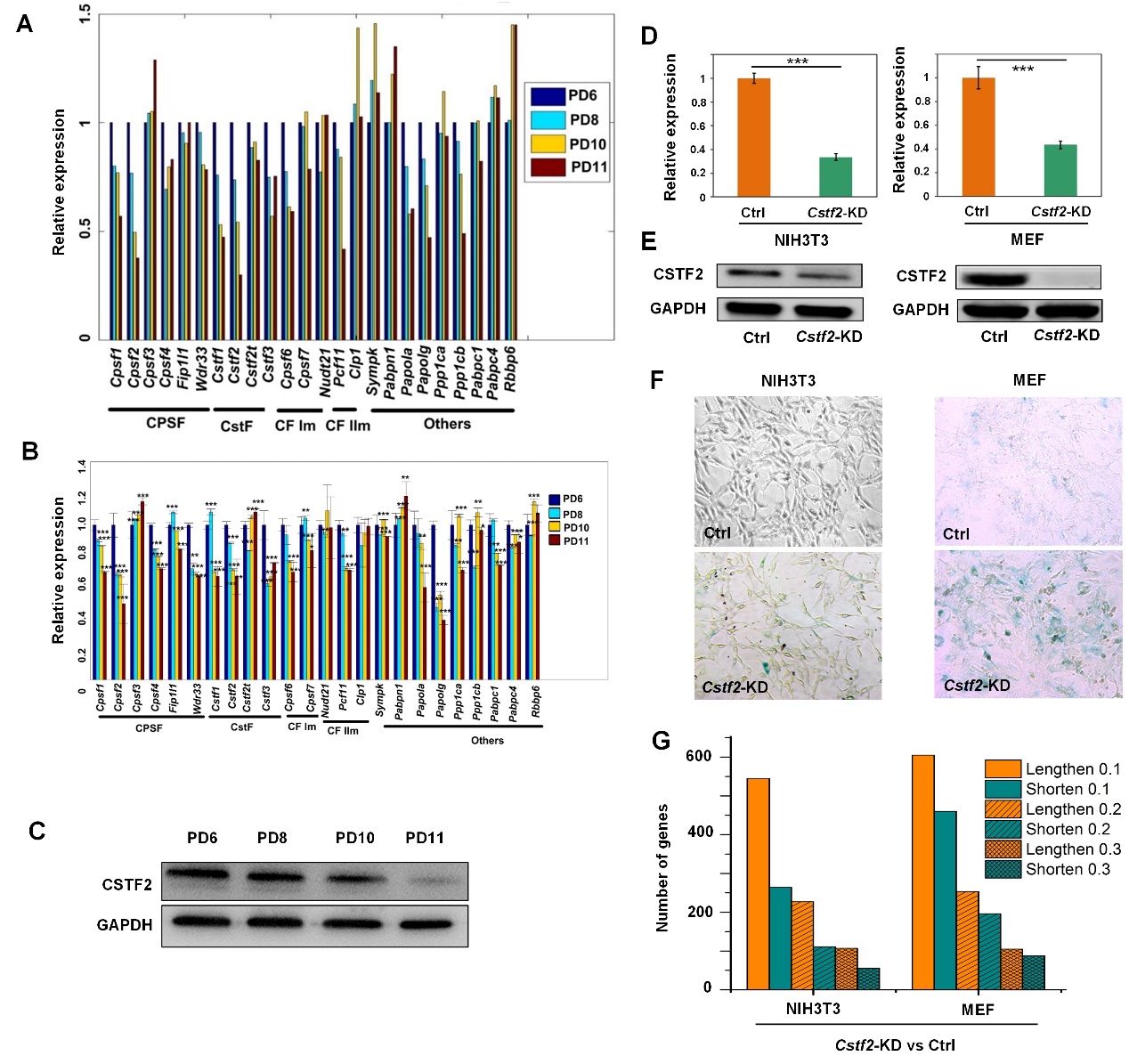


**Supplemental Figure S20. Gene expression of known cleavage and polyadenylation factors during replicative senescence of MEFs based on RNA-seq data (A) and qRT-PCR validation (B).** (***) P < 0.001, (**) P < 0.01 and (*) P < 0.05, two-tailed t-test.
