# Supplementary material for: 3′ UTR lengthening as a novel mechanism in regulating cellular senescence
Source: Genome Res. 2018 Mar;28(3):285–94. doi: 10.1101/gr.224451.117 (PMC5848608; doi:10.1101/gr.224451.117)
Supplement: Supplemental Material [file supp_gr.224451.117_Supplemental_Fig_S21.docx]

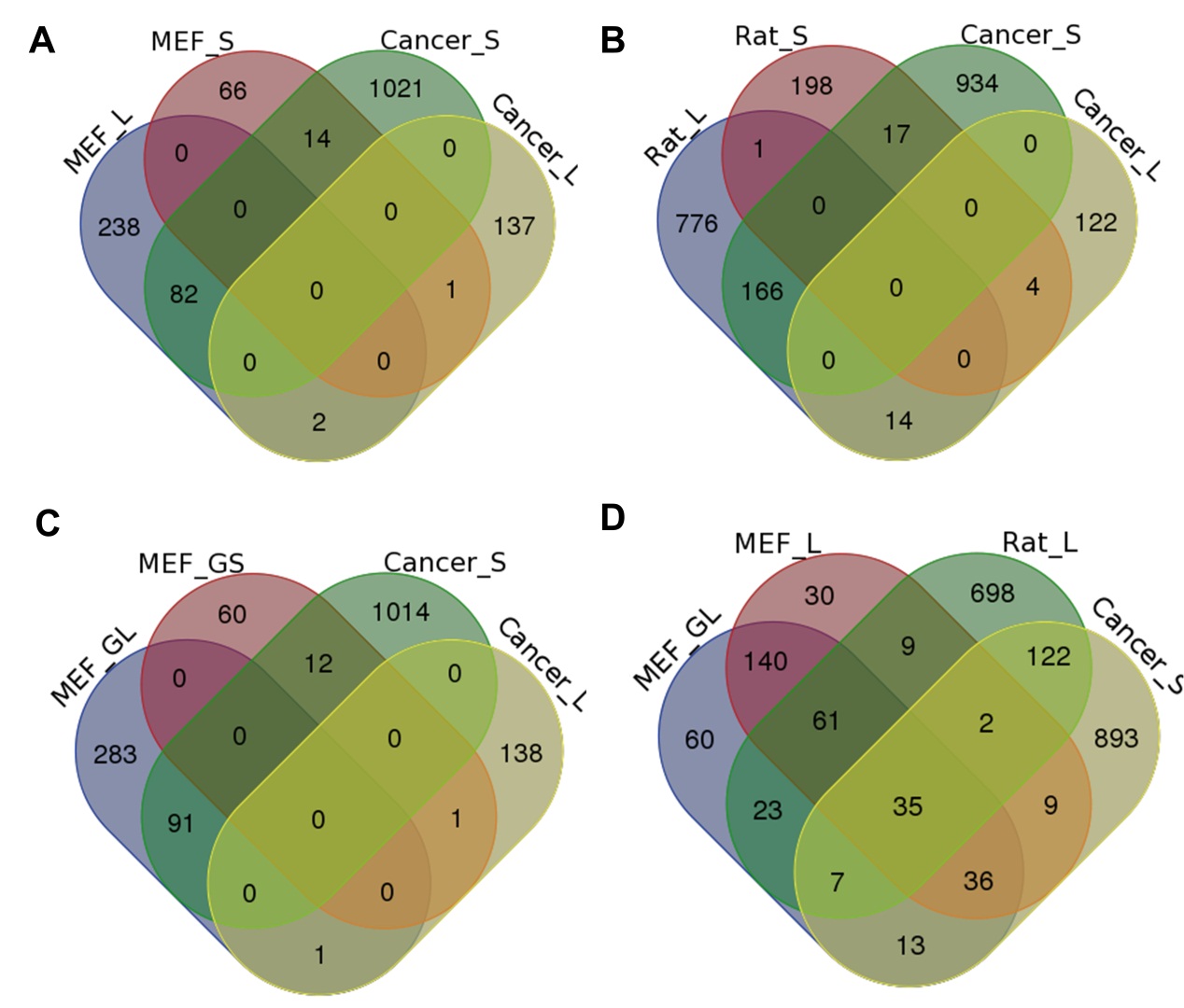


**Supplemental Figure S21. Comparison of genes that tended to use distal and proximal pAs between senescent and cancer cells.** (A) Venn diagram comparison among genes preferring distal (MEF_L) and proximal (MEF_S) pAs when comparing senescent MEFs (PD11) with young MEFs (PD6), and genes favoring distal (Cancer_L) and proximal (Cancer_S) pAs when comparing tumors and normal tissues identified by Xia et al. [3]. (B) Venn diagram comparison among genes preferring distal (Rat_L) and proximal (Rat_S) pAs when comparing VSMCs of old rat with young rat, Cancer_L, and Cancer_S. (C) Venn diagram comparison among genes gradually preferred to use distal (MEF_GL) and proximal (MEF_GS) pAs during replicative senescence of MEFs, Cancer_L, and Cancer_S. (D). Venn diagram comparison among MEF_L, Rat_L, MEF_GL, and Cancer_S. MEF_L, MEF_S, Rat_L, Rat_S, MEF_GL, and MEF_GS were identified by a linear trend test with the Benjamini-Hochberg (BH) false-discovery rate (FDR) at 5%.
