# Supplementary material for: 3′ UTR lengthening as a novel mechanism in regulating cellular senescence
Source: Genome Res. 2018 Mar;28(3):285–94. doi: 10.1101/gr.224451.117 (PMC5848608; doi:10.1101/gr.224451.117)
Supplement: Supplemental Material [file supp_gr.224451.117_Supplemental_Table_S1.docx]

**Supplemental Table 1. MEFs PA-seq** **reads mapping statistics.**

| **Sample** | **Total Reads** | **Mapped Read1** | **Read1 mapping rate** | **Mapped Read2** | **Read2 mapping rate** |
| --- | --- | --- | --- | --- | --- |
| G0 | 15,411,083 | 9,442,546 | 61.3% | 11,471,857 | 74.4% |
| PD6 | 9,423,643 | 4,478,586 | 47.5% | 5,689,182 | 60.4% |
| PD8 | 13,386,797 | 7,822,028 | 58.4% | 9,472,369 | 70.8% |
| PD10 | 8,779,338 | 3,818,641 | 43.5% | 4,799,825 | 54.7% |
| PD11 | 14,425,358 | 8,848,238 | 63.3% | 10,736,847 | 74.4% |

**Supplemental Table 2: 18,639 Refined pAs identified in this study and their assigned categories (available as a separate Excel file).**

**Supplemental Table 3. MEFs RNA-seq reads mapping statistics.**

| **Sample** | **Total Reads** | **Mapped Read1** | **Read1 mapping rate** | **Mapped Read2** | **Read2 mapping rate** |
| --- | --- | --- | --- | --- | --- |
| G0 | 15,809,150 | 13,997,188 | 88.5% | 15,809,150 | 88.6% |
| PD6 | 28,132,487 | 27,048,742 | 96.1% | 26,876,587 | 95.5% |
| PD8 | 25,659,880 | 24,321,086 | 94.8% | 24,175,499 | 94.2% |
| PD10 | 17,860,083 | 16,254,722 | 91.0% | 16,060,448 | 89.9% |
| PD11 | 16,396,313 | 14,101,035 | 86.0% | 13,980,835 | 85.3% |

**Supplemental Table 4: Summary 3,165 genes with APA regulation (available as a separate Excel file).**

**Supplemental Table 5. qRT-PCR primers used to validate genes with significant switch of APA usage.**

| **Gene** | **Common region primers  (from 5′ to 3′)** | **Alternative region primers  (from 5′ to 3′)** |
| --- | --- | --- |
| *Daam2* | GAGCGGGCCAATAAACAGG  CCTTTCTTCTAATCCCTGTCTCC | TTCAGGGGTTGGAAGGACAA  TTGCTTGTCTTCTGCAGCAG |
| *Anapc1* | CCTAGAAATGACTGCGGCAC  CATATGACACGTGGACAGCA | TGCCATGGGAGGACTTGAAA  GCTGTGCATGGTAAGACTGG |
| *Map3k7* | ACAGCAGGCTAATCAGGAGG  GGCAACAGACTCAGGAAAGG | GTTGGCACTCACTCACTTGG  GACCAGAGCTCACTTCCTGT |
| *Ccnh* | AGGAATGGACTGATGACGACC  CAGACATGCTTCCTACTTCCG | GCAAACCATGTCCTCCTGTG  TTGTAAGGGCTTCTGGAGGA |
| *Fbxo28* | TTCAGTTTTCTTTGGGGCCG  TGACAACTCTCTCCCGACAG | TGCCGATCCACACAGTCATA  AGACTCCACCATTCTCCAGC |
| *Gadd45b* | AGGTGGCCAGTTACTGTGAA  TTTAGGGGACAGCAACTCGA | GGAGACTGAGACTTTAGAGCCA  CTCCGCTGACTTATGCACAG |
| *Ube2b* | TCCAAACAGTCCAGCCAACA  TCCTTAAAACCCGTGGCACT | CCAGCTCTGTATTTGGGCCA  TCTCTGCCTTGCTCAAACCA |
| *Tceb2* | CATCGAGCCCTTTTCCAGC  GGGAAATGGGTCTCTAGGGG | AGGATTCTGGAGGCAGTGC  ACTTGGGGTTAAGAGTTCTGTG |
| *Ctsb* | AGGACAAATGCCACCTCTCA  GAGGACAGGACCAAGGAGAG | AGTATGAGTGCCAGGCCTTT  GGCTCTGTGGTAGTGGAAGT |
| *Acvr2a* | TGGAAAGCATGGATCTGGGA  TCCTTGATTTGGAGAGGGCC | CCTTGCCCAAATCTCCCATG  GTGCAAGTTCATGGGACCAA |
| *Sec61g* | TGCTTAAACGTGACTGCTTTTC  TTTACTTCATGCCCTTTCCCC | GGGTTGGCTGAGTCCTTCT  AGAAACAAAACACCACACAGC |
| *Lamc1* | TCCATCGAGAAGCCCTAGTG  AGTGATGGAGAGCAGCAGAG | ACTGTGGCCCTTTTCAATGT  AAAATGCCAAGTGTTGCGCT |
| *Cul1* | AACAGACGCCAATGCCATTT  TCCATTCAGACTCGCTCTCG | TAACAGCTGTCGTCTGAGGC  ACCAAATGCAACTTGTACAGAAA |

**Supplemental Table 6. PA-seq reads mapping statistics for biological replicate of MEF.**

| **Sample** | **Total Reads** | **Mapped Read1** | **Read1 mapping rate** | **Mapped Read2** | **Read2 mapping rate** |
| --- | --- | --- | --- | --- | --- |
| PD6 | 17,309,769 | 12,532,955 | 72.4% | 13,647,511 | 78.8% |
| PD11 | 26,553,217 | 17,115,583 | 64.5% | 18,725,415 | 70.5% |

**Supplemental Table 7. Rat PA-seq reads mapping statistics.**

| **Sample** | **Total Reads** | **Strand specific reads** | **Mapped Read1** | **Read1 mapping rate** | **Mapped Read2** | **Read2 mapping rate** |
| --- | --- | --- | --- | --- | --- | --- |
| 2 weeks | 26,122,246 | 20,468,514 | 8,818,888 | 43.1% | 11,694,741 | 57.1% |
| 2 years | 35,087,625 | 29,152,131 | 12,443,319 | 42.7% | 16,098,918 | 55.2% |

**Supplemental Table 8. List of genes with significant pA usage shift in replicative senescence of MEFs and aortic vascular smooth muscle cells of rats (VSMCs) at different ages. (Available as a separate Excel file)**

**Supplemental Table 9. Functional enrichment analysis for genes with significantly pA usage shift in replicative senescence of MEFs and aortic vascular smooth muscle cells of rats (VSMCs) at different ages. (Available as a separate Excel file).**

**Supplemental Table 10. Primer sequences for qRT-PCR, luciferase assay and shRNA (Available as a separate Excel file).**

**References in Supplemental Materials**
