# Supplementary material for: 3′ UTR lengthening as a novel mechanism in regulating cellular senescence
Source: Genome Res. 2018 Mar;28(3):285–94. doi: 10.1101/gr.224451.117 (PMC5848608; doi:10.1101/gr.224451.117)
Supplement: Supplemental Material [file supp_gr.224451.117_Supplemental_Table_S2.docx]

**Supplemental Table 2. MEFs RNA-seq reads mapping statistics.**

| **Sample** | **Total Reads** | **Mapped Read1** | **Read1 mapping rate** | **Mapped Read2** | **Read2 mapping rate** |
| --- | --- | --- | --- | --- | --- |
| G0 | 15,809,150 | 13,997,188 | 88.5% | 15,809,150 | 88.6% |
| PD6 | 28,132,487 | 27,048,742 | 96.1% | 26,876,587 | 95.5% |
| PD8 | 25,659,880 | 24,321,086 | 94.8% | 24,175,499 | 94.2% |
| PD10 | 17,860,083 | 16,254,722 | 91.0% | 16,060,448 | 89.9% |
| PD11 | 16,396,313 | 14,101,035 | 86.0% | 13,980,835 | 85.3% |
