# Supplementary material for: 3′ UTR lengthening as a novel mechanism in regulating cellular senescence
Source: Genome Res. 2018 Mar;28(3):285–94. doi: 10.1101/gr.224451.117 (PMC5848608; doi:10.1101/gr.224451.117)
Supplement: Supplemental Material [file supp_gr.224451.117_Supplemental_Table_S3.docx]

**Supplemental Table S3. PA-seq reads mapping statistics for biological replicate of MEFs.**

| **Sample** | **Total Reads** | **Mapped Read1** | **Read1 mapping rate** | **Mapped Read2** | **Read2 mapping rate** |
| --- | --- | --- | --- | --- | --- |
| PD6 | 17,309,769 | 12,532,955 | 72.4% | 13,647,511 | 78.8% |
| PD11 | 26,553,217 | 17,115,583 | 64.5% | 18,725,415 | 70.5% |
