# Supplementary material for: 3′ UTR lengthening as a novel mechanism in regulating cellular senescence
Source: Genome Res. 2018 Mar;28(3):285–94. doi: 10.1101/gr.224451.117 (PMC5848608; doi:10.1101/gr.224451.117)
Supplement: Supplemental Material [file supp_gr.224451.117_Supplemental_Table_S4.docx]

**Supplemental Table S4. Rat VSMCs PA-seq reads mapping statistics.**

| **Sample** | **Total Reads** | **Strand specific reads** | **Mapped Read1** | **Read1 mapping rate** | **Mapped Read2** | **Read2 mapping rate** |
| --- | --- | --- | --- | --- | --- | --- |
| 2 weeks | 26,122,246 | 20,468,514 | 8,818,888 | 43.1% | 11,694,741 | 57.1% |
| 2 years | 35,087,625 | 29,152,131 | 12,443,319 | 42.7% | 16,098,918 | 55.2% |
