# Supplementary material for: 3′ UTR lengthening as a novel mechanism in regulating cellular senescence
Source: Genome Res. 2018 Mar;28(3):285–94. doi: 10.1101/gr.224451.117 (PMC5848608; doi:10.1101/gr.224451.117)
Supplement: Supplemental Material [file supp_gr.224451.117_Supplemental_Methods.docx]

**Supplemental Methods**

**Cell isolation, cultivation and total RNA extraction**

Primary MEFs were isolated from embryos of a 12.5-14 day pregnant C57BL/6 mouse according to a previously described method ([Todaro and Green 1963](#_ENREF_7)). Cells were cultured in DMEM (Gibco, USA), supplemented with 10% fetal bovine serum (FBS; Gibco) and 1% penicillin/streptomycin (Gibco) at 37 °C in a humidified, 5% CO_2_ incubator. To establish the replicative senescence system, MEFs at 70%–80% confluence were transferred into new 100-mm dishes and cultured to the same 70%–80% confluence. This constituted one population doubling (PD). The number (n) of PDs was calculated using the equation PD = log_2_(Ne/Ns), where Ne is the number of cells at the end of cell culture and Ns is the number of cells seeded at the start of one passage ([Kim et al. 2013b](#_ENREF_3)). The number of PDs and the total culture period were monitored once every 3 days until MEFs completely stopped proliferating. TRIzol reagent (Invitrogen, USA) was used to extract total RNA from MEFs. Total RNA was also isolated using TRIzol reagent from rVSMCs from rats of different ages.

**SA-β-gal staining**

Culture medium was removed from cells grown in 12-well plates and the cells were washed once with 1 ml of 1× PBS and fixed with 0.5 ml of fixative solution for 10-15 min at room temperature. While the cells were in the fixative solution, the staining solution mix (staining solution, staining supplement, and 20 mg/ml of X-gal in DMSO) was prepared according to the manufacturer’s instructions (BioVision, cat. no. K320-250). After twice-washing with 1 ml of 1× PBS, cells were incubated with the aforementioned mix at 37 °C for 12 h or overnight. Cells were then observed under an inverted microscope (Leica).

**Cell cycle analysis**

MEFs were grown in 100-mm dishes and harvested with 1 ml of 0.25% trypsin upon reaching 70%–80% confluence. Cells were then washed twice with ice-cold 1× PBS, fixed with 70% ethanol for 2 hours (h) at 4 °C, washed again with ice-cold 1× PBS, permeabilized with 1% Triton X-100 plus RNase A (40 U/ml) and stained with propidium iodide (50 μg/ml) on a shaking platform at 37 °C. After staining, DNA content was measured on a FACS (fluorescence-activated cell sorting) Calibur HTS (Becton Dickinson). The percentages of diploid cells in the G1, S, and G2/M phases were analyzed using Summit version 5.0 software (Dako).

**Paired-end mapping and mRNA abundance estimation**

Paired-end reads from PA-seq were subjected to strand correction according to ‘TTT’ at the beginning of the reads, as previously described ([Ni et al. 2013](#_ENREF_5)). FastQC software was used for quality control of PA-seq and RNA-seq data (http://www.bioinformatics.bbsrc.ac.uk/projects/fastqc/). Processed raw data were then aligned to the mouse genome (version mm9) or rat genome (version rn5) using TopHat2 ([Kim et al. 2013a](#_ENREF_2)). Reads were mapped uniquely using the “-g 1” option. Bowtie ([Langmead et al. 2009](#_ENREF_4)) was used to achieve a good approximation for -r and --mate-std-dev parameters, which are required for TopHat2. Combined read counts of all pA clusters mapped to annotated PA, 3′ UTR, and extended 3′ UTR regions (number of Tags Per Million [TPM] reads) were used to evaluate the expression level of genes with APA. RNA-seq reads were also aligned to the genome in a similar way and Cufflinks was used to estimate mRNA abundance (fragments per kilobase of transcript per million mapped reads, FPKM) by following previously published instructions ([Trapnell et al. 2013](#_ENREF_8)). Genes expressed (FPKM ≥ 1 and TPM ≥ 1) in all passages were subjected to expression correlation analysis.

**Peak calling**

After mapping, pA site information was extracted. F-seq ([Boyle et al. 2008](#_ENREF_1)) was used for peak calling of pA sites using default parameters except that the parameter of feature length was set to 30. We resized the pA peaks to the shortest distance that contained 95% of reads falling into each corresponding peak according to our previous publication; the resized peaks were defined as pA clusters ([Ni et al. 2013](#_ENREF_5)). To filter out pA clusters derived from internal priming, we removed those with 15 ‘A’ in the 20 nucleotide region downstream peak mode (apex of the corresponding pA cluster) or with continuous 6 ‘A’ downstream peak mode. pA clusters with fewer than 20 supporting reads were removed. To further focus the analysis on robustly expressed APA events, we kept pA clusters that accounted for at least 10% of all the tags within respective genes in any sample and also low-expression pA clusters containing 5% of all the tags within respective genes in the majority of samples (80%); the resulting pA clusters were regarded as pA sites. RefSeq annotation was used for genomic location analysis. We classified the genome into eight groups and mapped the pA clusters to these groups using the following priority hierarchy: PA > 3′ UTR > extended 3′ UTR > exon > intron > 5′ UTR > transcription start site (TSS) > promoter. Specifically, PA was defined as an annotated pA site plus 10 bp upstream and 10 bp downstream. Extended_3′ UTR was the 3′ UTR plus 5 kb downstream according to a previous study ([Ni et al. 2013](#_ENREF_5)). Note that the peaks mapped to an extended 3′ UTR region should land in an intergenic region and not overlap with any known transcripts. TSS represents the exact transcription start site plus 10 bp upstream and 10bp downstream. The promoter was defined as the 250 bp upstream TSS, which should not overlap with any known transcripts.

**Conservation analysis surrounding pA sites**

Conservation scores in the 400 nt surrounding different types of pA sites were downloaded from the UCSC Genome Browser. The conservation score was measured according to phastCons scores based on 100 vertebrates ([Thomas et al. 2003](#_ENREF_6)).

**Comparison of PA-seq data with PolyA_DB**

PolyA_DB2 was downloaded from Bin Tian’s website (http://exon.umdnj.edu/polya_db). LiftOver (http://genome.ucsc.edu/cgi-bin/hgLiftOver) was applied to convert mm5 to mm9 for pA sites in PolyA_DB2. Due to the imprecise nature of cleavage by CPSF-73 or the fuzzy definition of pA sites by their surrounding *cis* elements, cleavages may occur at different positions within a small sequence window. Therefore, we considered pA sites identified by PolyA_DB2 to be the same pA sites identified in our data if they are located 10 bp upstream or 10 bp downstream of a pA site.

**qRT-PCR and western blotting**

MEFs were harvested at 70%–80% confluence. Total RNA and protein were isolated with TRIzol reagent according to manufacturer’s instructions (Invitrogen). For qRT-PCR analysis, RNA was reverse-transcribed into cDNA using random primers, and then mRNA levels were quantified by qPCR and normalized to that of *Gapdh* (Roche LightCycler). For western blot analysis, proteins were subjected to SDS-PAGE, transferred to nitrocellulose membranes, and incubated with primary antibodies of interest overnight at 4 °C. Blots were then washed three times in PBST (1× PBS + 0.1% Tween-20) and incubated with secondary antibodies (IRDye800CW goat anti-mouse/rabbit, 1:10000, cat. no. 926-32210/11, LI-COR) for 1-2 h at room temperature. Blots were then washed three times with PBST before visualization under an Odyssey infrared imaging system (Odessey, LI-COR).

**Validation of pA site usage preference by qRT-PCR**

RNAs were reverse-transcribed with oligo(dT) primer, followed by PCR with two pairs of primers (proximal and distal) targeting different regions of the cDNAs. Specifically, the region targeted by the proximal pair is common to both APA isoforms and the region targeted by the distal pair is unique to the longer isoform. qPCR signals from the proximal and distal pairs of primers were compared to indicate the relative expression of the two isoforms.

**RIP-PCR**

The TRA2B coding sequence was cloned into pCDNA3.1 vector containing the Myc tag. Forty-eight hours after transfection, 1 × 10^7^ primary MEFs were lysed and incubated with Myc antibody (Myc, 1:500, cat. no. M20002, abmart). RNA-protein complexes were immunoprecipitated with protein G beads and RNA was extracted using TRIzol reagent.

**mRNA stability and luciferase assays**

*Renilla* luciferase in psiCHECK-2 Vector (Promega, cat. no. C8021) was used as a primary reporter gene, and the 3′ UTR of interest was cloned downstream of the *Renilla* luciferase translational stop codon. The firefly reporter cassette served as an intra-plasmid transfection normalization reporter. NIH3T3 cells were seeded into a 24-well plate at a density of 15,000 cells/well, and two kinds of mouse *Rras2* 3′ UTR (a short 3′ UTR, and a long 3′ UTR with mutated proximal PAS) were separately sub-cloned into psiCHECK-2 vector using the XhoI and PmeI restriction enzyme sites. After overnight incubation, the cells were treated with a transfection mixture consisting of 50 μl serum-free medium and 1 μl Lipofectin reagent (Life Technologies, cat. no. E2431), together with 0.5 μg psiCHECK-2 vector:short, 0.5 μg psiCHECK-2 vector:long, or 0.5 μg psiCHECK-2 vector:mutant. Twenty-four hours post-transfection, *Renilla* and firefly luciferase activities were measured using the Dual-Luciferase Reporter 1000 Assay System (Promega).

**References**

Boyle AP, Guinney J, Crawford GE, Furey TS. 2008. F-Seq: a feature density estimator for high-throughput sequence tags. *Bioinformatics* 24(21): 2537-2538.

Kim D, Pertea G, Trapnell C, Pimentel H, Kelley R, Salzberg SL. 2013a. TopHat2: accurate alignment of transcriptomes in the presence of insertions, deletions and gene fusions. *Genome Biol* 14(4): R36.

Kim YM, Byun HO, Jee BA, Cho H, Seo YH, Kim YS, Park MH, Chung HY, Woo HG, Yoon G. 2013b. Implications of time-series gene expression profiles of replicative senescence. *Aging cell* 12(4): 622-634.

Langmead B, Trapnell C, Pop M, Salzberg SL. 2009. Ultrafast and memory-efficient alignment of short DNA sequences to the human genome. *Genome Biol* 10(3): R25.

Ni T, Yang Y, Hafez D, Yang W, Kiesewetter K, Wakabayashi Y, Ohler U, Peng W, Zhu J. 2013. Distinct polyadenylation landscapes of diverse human tissues revealed by a modified PA-seq strategy. *BMC Genomics* 14: 615.

Thomas JW, Touchman JW, Blakesley RW, Bouffard GG, Beckstrom-Sternberg SM, Margulies EH, Blanchette M, Siepel AC, Thomas PJ, McDowell JC et al. 2003. Comparative analyses of multi-species sequences from targeted genomic regions. *Nature* 424(6950): 788-793.

Todaro GJ, Green H. 1963. Quantitative studies of the growth of mouse embryo cells in culture and their development into established lines. *J Cell Biol* 17: 299-313.

Trapnell C, Hendrickson DG, Sauvageau M, Goff L, Rinn JL, Pachter L. 2013. Differential analysis of gene regulation at transcript resolution with RNA-seq. *Nat Biotechnol* 31(1): 46-53.
